# Preparation of GO/SiO_2_/PEA as a new solid base catalyst for the green synthesis of some spirooxindole derivatives†

**DOI:** 10.1039/d1ra02850b

**Published:** 2021-06-21

**Authors:** Mahla Toorbaf, Leila Moradi

**Affiliations:** Department of Organic Chemistry, Faculty of Chemistry, University of Kashan P.O. Box 8731753153 Kashan I. R. Iran l_moradi@kashanu.ac.ir

## Abstract

Efficient and green one pot multi component synthesis of some spirooxindole derivatives in the presence of graphene oxide functionalized with 2-(1-piperazinyl) ethylamine (GO/SiO_2_/PEA) as a solid base catalyst was studied. GO/SiO_2_/PEA has been obtained through a two step reaction and characterized by Fourier transform infrared spectroscopy (FTIR), field emission scanning electron microscopy (FE-SEM), energy-dispersive X-ray spectroscopy (EDX), thermo gravimetric analysis (TGA), Raman spectroscopy and X-ray diffraction (XRD). Green reaction conditions, short reaction times, reusable catalyst and a high to excellent yield of products are some of the advantageous of the presented method.

## Abstract 2

Efficient and green one pot multi component synthesis of some spirooxindole derivatives in the presence of graphene oxide functionalized with 2-(1-piperazinyl) ethylamine (GO/SiO_2_/PEA) as a solid base catalyst was studied.

## Graphical abstract

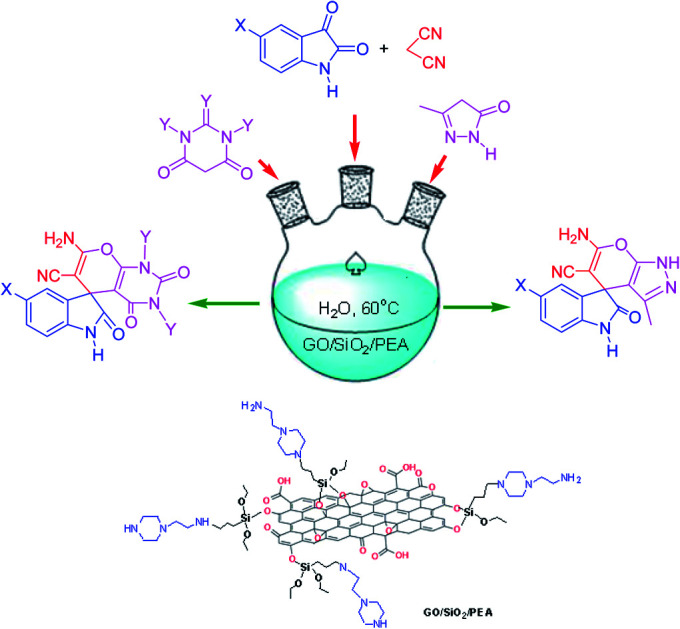

## Introduction

Multicomponent reactions (MCRs) allow quick and easy access to a large group of organic compounds.^1–3^ Three or more reactants reacted to form the products consisting of all raw materials.^4^ Because these reactions do not require intermediate separation, their efficiency increases dramatically. Simple conditions, reduction of byproducts, easy separation, energy saving and short reaction times are the benefits of these reactions.^5^

Recently, green approaches in designing the synthetic methods have attracted considerable attention. Green chemistry means the design of chemical products and processes that reduce or eliminate the use and production of harmful substances to human health and the environment. The goals of this approach are the implementation and sustainable development of chemistry and technology in the chemical industry, academia and government.^6^ Green chemistry is usually presented as a set of twelve principles suggested by Anastas and Warner, including guidelines for the chemists that work on the synthesis of materials, new chemicals, and new technological processes.^7,8^

Spiroxindoles as a kind of polycyclic compounds, play an important role in the medical fields. Different derivatives of spiroxindoles are seriously on the agenda of scientists. On the other hand, with the change of precursors and methods of preparation of these compounds, new features of them are revealed in the medical and pharmaceutical fields. The antibacterial, anticancer, antitumour, antifungal and antimicrobial activities of these molecules have been demonstrated^9–12^ therefore; it is desirable to develop simple and highly efficient methods for the preparation of spiroxindoles. Some of catalysts for synthesis of these compounds are include: piperidin^13^, Et_3_N,^14^ MNPs-guanidne,^15^ Na_2_CaP_2_O_7_,^16^l-proline,^17^ meglumine,^18^ SnO_2_ (ref. 19) and CuO nanoparticles^20^ and also 4-dimethylaminopyridine.^21^

In recent years, supported catalysts as green and reusable catalysts have received much attention in the design of chemical syntheses. Graphene oxide (GO) and reduced graphene oxide has been used as catalyst support due to high surface area, easily available, low cast and high activity.^22–24^ Graphene is a two-dimensional sheet of carbon atoms in a hexagonal configuration in which atoms are bonded to the sp^2^ hybrid. This structure is the newest member of the multi dimensional graphite carbon family.^25,26^ Graphene oxide is a nanocarbon material with a honeycomb-like carbon plate which has epoxide, hydroxyl and carboxyl functional groups on its surfaces.

**Scheme 1:**
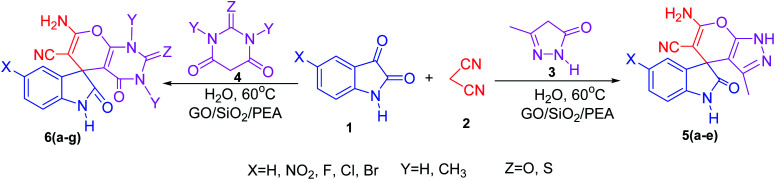
Synthesis of spirooxindoles in the presence of GO/SiO_2_/PEA.

In presented method, we try to design a new heterogeneous catalyst based on graphene oxide. In fact, an amino group has been chemically attached on graphene oxide surfaces through SiO_2_ linker. Obtained basic catalyst was used in green synthesis of some spirooxindoles (Scheme 1). Results show the high efficiency of catalyst as well as short reaction times with high to excellent yield of products.

## Experimental section

### Materials and methods

Chemical reagents were purchased from the Merck and Aldrich Company. FT-IR spectra were obtained using KBr pellets on a Perkin Elmer 781 spectrophotometer and on an impact 400 Nicolet FTIR spectrophotometer. ^1^H NMR and ^13^C NMR spectra were measured with DMSO-d_6_ solution and tetramethylsilane as internal standard on a Bruker DRX-400 spectrometer. Thermo gravimetric analysis (TGA) was performed on a mettle TA4000 system TG-50 at a heating rate of 10 K min^−1^ under N_2_ atmosphere. Raman spectroscopy was provided using FRA/106/S. To investigate the morphology of the MWCNTs and prepared catalyst, FE-SEM images and EDX analysis visualized by a Sigma ZEISS, Oxford Instruments Field Emission Scanning Electron Microscope. TEM images was provided using a Philips CM 120, Netherlands microscope with an accelerating voltage of 150 kV.

### Preparation of graphene oxide (GO)

GO was synthesized from graphite using the Hummers method.^27^ Briefly in a typical procedure, 1 g graphite, 15 ml H_3_PO_4_ and 135 ml H_2_SO_4_ were mixed. 6.6 g KMnO_4_ was slowly added while stirring, and the rate of addition was controlled to prevent the mixture temperature exceeding from 5 °C. Then, to remove the excess amounts of KMnO_4_, 3.5 ml of H_2_O_2_ was slowly added and stirred for 10 minutes. After left the mixture to cool down, 25 ml of HCl and 15 ml of (deionized water) DI water was added and centrifuged. The supernatant was decanted and the residuals was then rewashed again with HCl and DI water for 3 times and centrifuged. Obtained washed GO was dried at 90 °C for 24 hours to produce the powder of GO.

### Preparation of graphene oxide functionalized with 2-(1-piperazinyl) ethylamine (GO/SiO_2_/PEA)

**Scheme 2:**
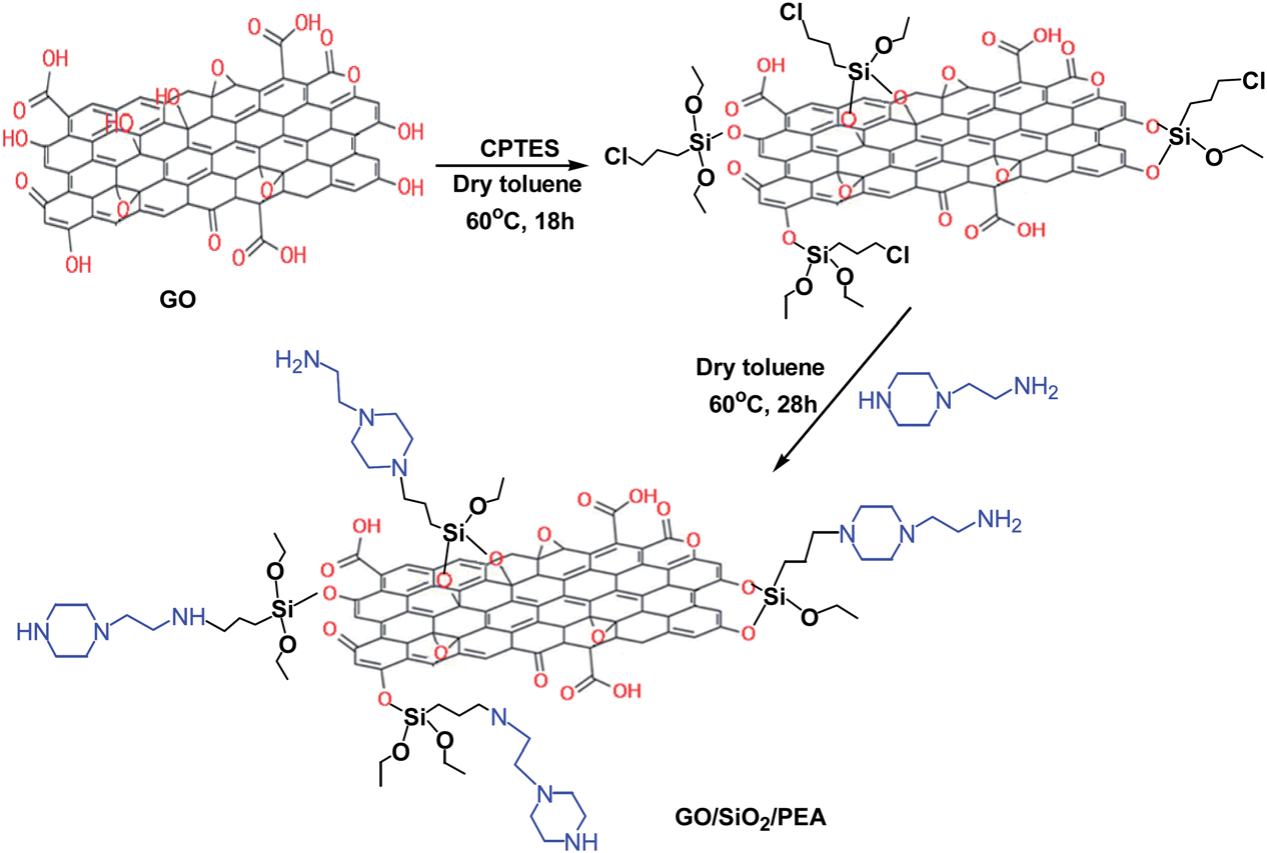
Preparation of GO/SiO_2_/PEA catalyst.

In a balloon equipped with a magnetic stirrer and a nitrogen gas conduction system, 1 g of graphene oxide, 100 ml of dry toluene and 1 ml of (3-chloropropyl) triethoxyethane were mixed and refluxed at 60 °C for 18 h. After the completion of this step, the content of the balloon was filtered and obtained solid (GO/CPTES) washed with EtOH and dried at 80 °C for 16 hours.^28^ For preparation of GO/SiO_2_/PEA, 0.5 g of GO/CPTES was dispersed in 15 ml of dry toluene for 10 min by ultrasonication. Then 0.5 ml of 2-(1-piperazinyl) ethylamine (PEA) was added to the mixture and refluxed for 28 hours. After the time had elapsed, the mixture was filtered and the final product was washed with ethanol and dried at 80 °C for 12 h (Scheme 2).

### General procedure for the preparation of spiro derivatives

For the synthesis of spiro [indulin-3,4′-pyrano[2,3-*c*]pyrazole]-5′-carbonitrile derivatives (5a–e), a mixture of ethyl acetoacetate (1 mmol) and hydrazine hydrate (1 mmol) were stirred by magnetic stirrer in 5 ml of water for 15 minutes at 60 °C. After the formation of 3-methyl-2-pyrazoline-5-one as a white precipitate, isatin (1 mmol) and malononitrile (1 mmol) and also catalyst (0.015 g) were added and reaction was continued to complete (followed by thin-layer chromatography). After the reaction was completed, the precipitate was dissolved in ethanol and the catalyst was separated by filtration. Evaporation of solvent from the filtrate, resulted the crud product and for further purification, recrystallized from ethanol (Scheme 1).

In order to the synthesis of 7-amino-2,4-dihydro [indoline-3,5-pyrano(2,3-*d*)pyrimidine]-6-carbonitriles (6a–g), a mixture of isatin (1 mmol), malononitrile (1 mmol) and barbituric acid derivative (1 mmol) in the presence of 0.015 g of catalyst and 5 ml of water was stirred by magnetic stirrer at 60 °C for 18 min. After the completion of reaction was complete, the separation and purification of products was the same as previous synthetic method.

### Spectral data

#### 6′-Amino-3′-methyl-2-oxo-1′*H*-spiro[indoline-3,4′-pyrano[2,3-*c*]pyrazole]-5′-carbonitrile (5a)

Mp 232–234 °C. FTIR (KBr) *ν*_max_: 3337 (NH), 3132 (NH), 2182 (C=N), 1711 (C=O), 1496 (C–H, Ar) cm^−1^; ^1^H NMR (400 MHz, DMSO-d_6_) *δ*_H_: 1.51 (3H, s, CH_3_), 6.88 (1H, d, *J* = 7.0 Hz, ArH), 6.96 (2H, m, ArH), 7.22 (2H, s, NH_2_), 7.24 (1H, s, ArH), 10.59 (1H, s, NH), 12.27 (1H, s, NH) ppm; ^13^C NMR (100 MHz, DMSO-d_6_) *δ*: 12.5, 47.1, 58.5, 111.1, 112.5, 116.2, 122.1, 125.6, 127.7, 140.7, 162.6, 166.9, 175.3 ppm. Anal. calcd for C_15_H_11_N_5_O_2_: C, 61.43; H, 3.78; N, 23.88%. Found: C, 61.36; H, 3.75; N, 23.82%.

#### 6′-Amino-3′-methyl-2-oxo-1′*H*-spiro[5-nitro-indoline-3,4′-pyrano[2,3-*c*]pyrazole]-5′-carbonitrile (5b)

Mp 226–228 °C. FTIR (KBr) *ν*_max_: 3419–3337 (NH), 3174 (NH), 2187 (C=N), 1720 (C=O), 1498 (C–H, Ar) cm^−1^; ^1^H NMR (400 MHz, DMSO-d_6_) *δ*_H_: 1.57 (3H, s, CH_3_), 7.12 (1H, d, *J* = 7.0 Hz, ArH), 7.41 (2H, s, NH_2_), 7.90 (1H, s, ArH), 8.21 (1H, d, *J* = 8.0 Hz, ArH), 11.36 (1H, s, NH), 12.40 (1H, s, NH) ppm; ^13^C NMR (100 MHz, DMSO-d_6_) *δ*_C_: 9.8, 48.3, 55.4, 95.2, 110.8, 119.8, 121.6, 127.3, 134.6, 136.5, 144.5, 148.9, 156.7, 164.1, 178.9 ppm; anal. calcd for C_15_H_10_N_6_O_4_: C, 53.25; H, 2.96; N, 24.85%. Found: C, 53.22; H, 3.12; N, 24.79%.

#### 6′-Amino-3′-methyl-2-oxo-1′*H*-spiro[5-fluoro-indoline-3,4′-pyrano[2,3-*c*]pyrazole]-5′ carbonitrile (5c)

Mp 262–265 °C. FTIR (KBr) *ν*_max_: 3409 (NH), 3220 (NH), 2186 (C=N), 1706 (C=O), 1490 (C–H, Ar) cm^−1^; ^1^H NMR (400 MHz, DMSO-d_6_) *δ*_H_: 1.56 (3H, s, CH_3_), 6.90 (1H, s, ArH), 6.97 (1H, d, *J* = 6.0 Hz, ArH), 7.07 (1H, d, *J* = 7.0 Hz, ArH), 7.28 (2H, s, NH_2_), 10.62 (1H, s, NH), 12.31 (1H, s, NH) ppm; ^13^C NMR (100 MHz, DMSO-d_6_) *δ*_C_: 9.4, 48.3, 55.3, 95.4, 111.0, 116.2, 119.1, 134.9, 135.2, 138.3, 147.6, 155.8, 157.8, 163.3, 178.2 ppm; anal. calcd for C_15_H_10_FN_5_O_2_: C, 57.82; H, 3.21; N, 22.49%. Found: C, 57.79; H, 3.23; N, 22.51%.

#### 6′-Amino-3′-methyl-2-oxo-1′*H*-spiro[5-chloro-indoline-3,4′-pyrano[2,3-*c*]pyrazole]-5′-carbonitrile (5d)

Mp 232–234 °C. FTIR (KBr) *ν*_max_: 3346 (NH), 3137 (NH), 2182 (C=N), 1714 (C=O), 1487 (C–H, Ar) cm^−1^; ^1^H NMR (400 MHz, DMSO-d_6_) *δ*_H_: 1.57 (3H, s, CH_3_), 6.90 (1H, d, *J* = 6.0 Hz, ArH), 7.12 (1H, d, *J* = 7.0 Hz, ArH), 7.29 (1H, s, ArH), 7.29 (2H, s, NH_2_), 10.74 (1H, s, NH), 12.33 (1H, s, NH) ppm; ^13^C NMR (100 MHz, DMSO-d_6_) *δ*_C_: 9.4, 48.1, 55.1, 95.2, 112.0, 119.1, 125.1, 127.1, 129.5, 135.6, 141.3, 155.7, 163.3, 177.9 ppm; anal. calcd for C_15_H_10_ClN_5_O_2_: C, 54.88; H, 3.05; N, 21.34%. Found: C, 54.89; H, 3.09; N, 21.41%.

#### 6′-Amino-3′-methyl-2-oxo-1′*H*-spiro[5-bromo-indoline-3,4′-pyrano[2,3-*c*]pyrazole]-5′-carbonitrile (5e)

Mp 248–250 °C. FTIR (KBr) *ν*_max_: 3346 (NH), 3141 (NH), 2182 (C=N), 1713 (C=O), 1498 (C–H, Ar) cm^−1^; ^1^H NMR (400 MHz, DMSO-d_6_) *δ*_H_: 1.56 (3H, s, CH_3_), 6.86 (1H, d, *J* = 6.0 Hz, ArH), 7.22 (1H, s, ArH), 7.30 (2H, s, NH_2_), 7.41 (1H, d, *J* = 7.0 Hz, ArH), 10.76 (1H, s, NH), 12.34 (1H, s, NH) ppm; ^13^C NMR (100 MHz, DMSO-d_6_) *δ*_C_: 9.4, 48.0, 55.0, 95.2, 112.2, 114.7, 119.5, 128.0, 132.2, 135.2, 135.5, 141.3, 155.6, 163.1, 178.2 ppm; anal. calcd for C_15_H_10_BrN_5_O_2_: C, 48.41; H, 2.71; N, 18.82%. Found: C, 48.44; H, 2.65; N, 18.74%.

#### 7′-Amino-2,4′-dioxo-2′-thioxo-1′,2′,3′,4′-tetrahydrospiro[indoline-3,5′-pyrano[2,3-*d*]pyrimidine]-6′-carbonitrile (6a)

Mp 281–283 °C. FTIR (KBr) *ν*_max_: 3321 (NH_2_), 2197 (C=N), 1679 (C=O amide), 1249 (C=O) cm^−1^; ^1^H NMR (400 MHz, DMSO-d_6_) *δ*_H_: 6.77(1H, d, *J* = 6.0 Hz, ArH), 6.88 (1H, t, *J* = 6.0 Hz ArH), 7.16 (2H, t, *J* = 7.0 Hz, ArH), 7.45 (2H s, NH_2_), 10.56 (1H, s, NH), 12.51 (1H, s, NH) ppm; ^13^C NMR (100 MHz, DMSO-d_6_) *δ*_c_: 45.1, 57.5, 85.2, 112.4, 119.6, 123.7, 124.3, 129.1, 133.7, 142.6, 163.9, 166.1, 167.9, 176.6, 183.2 ppm; anal. calcd for C_15_H_9_N_5_O_2_S: C, 45.79; H, 2.29; N, 17.81%. Found: C, 45.81; H, 2.27; N, 17.92%.

#### 7′-Amino-5-fluoro-1,1′,2,2′,3′,4′-hexahydro-2,4′-dioxo-2′-thioxospiro[indole-3,5′-pyrano[2,3-*d*]pyrimidine]-6′-carbonitrile (6b)

Mp 299–301 °C. FTIR (KBr) *ν*_max_: 3367 (NH_2_), 3163 (NH), 2199 (C=N), 1684 (C=O amide), 1258 (C=O), 1485 (C=C), 1184–1341 (C–O) cm^−1^; ^1^H NMR (400 MHz, DMSO-d_6_) *δ*_H_: 6.76 (1H, t, *J* = 6.0 Hz ArH), 6.95 (1H, t, *J* = 6.0 Hz, ArH), 7.18 (1H, d, *J* = 7.0 Hz, ArH), 7.44 (2H s, NH_2_), 10.53 (1H, s, NH), 12.40 (1H, s, NH) ppm; ^13^C NMR (100 MHz, DMSO-d_6_) *δ*_C_: 47.1, 57.0, 91.2, 110.0, 112.3, 115.1, 116.8, 134.7, 138.4, 152.9, 157.1, 158.2, 159.2, 174.1, 177.4 ppm; anal. calcd for C_15_H_8_FN_5_O_3_S: C, 50.42; H, 2.24; N, 19.61%. Found: C, 50.44; H, 2.22; N, 19.59%.

#### 7′-Amino-5-chloro-1,1′,2,2′,3′,4′-hexahydro-2,4′-dioxo-2′-thioxospiro[indole-3,5′-pyrano[2,3-*d*]pyrimidine]-6′-carbonitrile (6c)

Mp 289–291 °C. FTIR (KBr) *ν*_max_: 3357 (NH_2_), 2197 (C=N), 1681 (C=O amide), 1222 (C=O), 1476 (C=C), 1302–1172 (C–O) cm^−1^; ^1^H NMR (400 MHz, DMSO-d_6_) *δ*_H_: 6.77 (1H, d, *J* = 6.0 Hz, ArH), 7.19 (1H, d, *J* = 7.0 Hz, ArH), 7.36 (1H, s, ArH), 7.45 (2H s, NH_2_), 10.63 (1H, s, NH), 12.39 (1H, s, NH) ppm; ^13^C NMR (100 MHz, DMSO-d_6_) *δ*_C_: 48.3, 56.8, 91.0, 110.8, 116.9, 124.4, 126.1, 128.6, 135.2, 141.1, 153.3, 158.3, 160.1, 174.3, 177.1 ppm; anal. calcd for C_15_H_8_ClN_5_O_3_S: C, 48.15; H, 2.14; N, 18.72%. Found: C, 48.14; H, 2.18; N, 18.81%.

#### 7′-Amino-5-bromo-1,1′,2,2′,3′,4′-hexahydro-2,4′-dioxo-2′-thioxospiro[indole-3,5′-pyrano[2,3-*d*]pyrimidine]-6′-carbonitrile (6d)

Mp 238–240 °C. FTIR (KBr) *ν*_max_: 3350 (NH_2_), 2199 (C=N), 1691 (C=O amide), 1213 (C=O), 1339–1169 (C–O) cm^−1^; ^1^H NMR (400 MHz, DMSO-d_6_) *δ*_H_: 6.74 (1H, d, *J* = 6.0 Hz, ArH), 7.32 (1H, t, *J* = 7.0 Hz, ArH), 7.50 (1H, s, ArH), 7.50 (2H s, NH_2_), 10.68 (1H, s, NH), 12.54 (1H, s, NH) ppm; ^13^C NMR (100 MHz, DMSO-d_6_) *δ*_C_: 47.3, 57.3, 91.5, 111.7, 114.1, 117.3, 127.5, 131.8, 137.1, 142.5, 153.8, 158.8, 160.0, 174.8, 177.5 ppm; anal. calcd for C_15_H_8_BrN_5_O_3_S: C, 43.06; H, 1.91; N, 16.74%. Found: C, 43.11; H, 1.89; N, 16.69%.

#### 2-Amino-6,8-dimethyl-5,7-dioxospiro[5′-chloro-(3′*H*)-indol-3′,4,4(*H*)-5,6,7,8-tetrahydropyrano(2,3-*d*)pyrimidine]-(1′*H*)-2′-one-3-carbonitrile (6e)

Mp 248–250 °C. FTIR (KBr) *ν*_max_: 3407 (NH_2_), 2197 (C=N), 1681 (C=O amide), 1245 (C=O), 1499 (C=C), 1302–1172 (C–O) cm^−1^; ^1^H NMR (400 MHz, DMSO-d_6_) *δ*_H_: 3.02 (3H, s, CH_3_), 3.35 (3H, s, CH_3_), 6.79 (1H, d, *J* = 6.0 Hz, ArH), 7.18 (1H, d, *J* = 7.0 Hz, ArH), 7.27 (1H s, ArH), 7.64 (2H, s, NH_2_), 10.63 (1H, s, NH) ppm; ^13^C NMR (100 MHz, DMSO-d_6_) *δ*_c_: 28.2, 29.7, 48.1, 56.9, 86.1, 113.5, 117.2, 118.0, 126.4, 130.6, 136.3, 140.6, 151.1, 152.6, 157.9, 165.9, 178.1 ppm; anal. calcd for C_17_H_12_ClN_5_O_4_: C, 31.10; H, 3.11; N, 18.13%. Found: C, 31.12; H, 3.09; N, 18.15%.

#### 2-Amino-6,8-dimethyl-5,7-dioxospiro[5′-fluoro-(3′*H*)-indol-3′,4,4(*H*)-5,6,7,8-tetrahydropyrano(2,3-*d*)pyrimidine]-(1′*H*)-2′-one-3-carbonitrile (6f)

Mp 276–278 °C. FTIR (KBr) *ν*_max_: 3484 (NH_2_), 2197 (C=N), 1681 (C=O amide), 1288 (C=O), 1486 (C=C) cm^−1^; ^1^H NMR (400 MHz, DMSO-d_6_) *δ*_H_: 3.01 (3H, s, CH_3_), 3.46 (3H, s, CH_3_), 6.77 (1H, m, ArH), 6.97 (1H, t, *J* = 6.0 Hz ArH), 7.07 (1H, s, ArH), 7.62 (2H, s, NH_2_), 10.52 (1H, s, NH), 12.50 (1H, s, NH) ppm; ^13^C NMR (100 MHz, DMSO-d_6_) *δ*_C_: 28.2, 30.1, 47.8, 56.9, 85.4, 87.1, 112.3, 117.2, 132.6, 136.9, 137.5, 142.3, 150.1, 153.1, 159.1, 161.0, 178.3 ppm; anal. calcd for C_17_H_12_FN_5_O_4_: C, 32.49; H, 3.24; N, 18.95%. Found: C, 32.46; H, 3.28; N, 18.91%.

#### 7′-Amino-1,1′,2,2′,3′,4′-hexahydro-1′,3′-dimethyl-2,2′,4′-trioxospiro[indole-3,5′-pyrano[2,3-*d*]pyrimidine]-6′-carbonitrile (6g)

Mp 299–301 °C. FTIR (KBr) *ν*_max_: 3428 (NH_2_), 3317 (NH), 2924 (CH_2_), 2000 (C=N), 1693 (C=O amide), 1652 (C=O) cm^−1^; ^1^H NMR (400 MHz, DMSO-d_6_) *δ*_H_: 3.01 (3H, s, CH_3_), 3.71 (3H, s, CH_3_), 6.95 (1H, d, *J* = 6.0 Hz ArH). 7.28 (2H, t, *J* = 7.0 Hz, ArH), 7.43 (1H, t, *J* = 7.0 Hz, ArH), 7.13 (2H, s, NH_2_) ppm; ^13^C NMR (100 MHz, DMSO-d_6_) *δ*_C_: 27.4, 29.6, 48.0, 57.5, 87.3, 109.6, 117.0, 121.8, 123.9, 128.4, 133.5, 142.2, 150.0, 152.1, 158.3, 159.2, 177.5 ppm; anal. calcd for C_17_H_13_N_5_O_4_: C, 34.16; H, 3.70; N, 19.94%. Found: C, 43.14; H, 3.73; N, 19.97%.

## Results and discussions

### Characterization of catalyst

We have established that GO/SiO_2_/PEA is an efficient, reusable and heterogeneous solid base catalyst for the synthesis of spirooxindole derivatives. Presented method has several advantages including excellent yield of products, green reaction conditions, reusability of catalyst and the simple work-up process. Prepared catalyst was characterized using various methods.

**Fig. 1:**
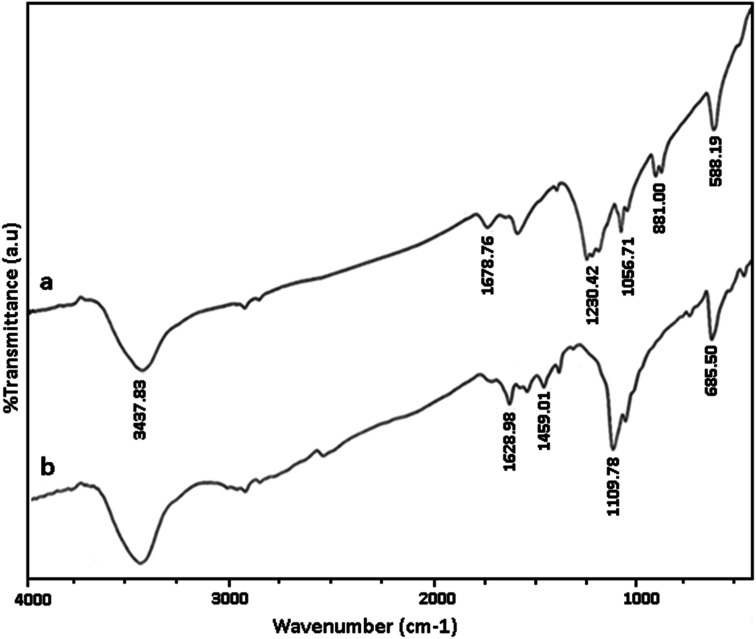
FT-IR of: (a) GO, (b) GO/SiO_2_/PEA.

Fig. 1a and b shows the FTIR spectra of GO and GO/SiO_2_/PEA. In the FT-IR spectrum, of graphene oxide, the peak at 1578 cm^−1^ corresponds to carbon–carbon double bonds. The absorption peaks in the 1057, 1678 and 3437 cm^−1^ are related to the vibration of C–O, carbonyl and hydroxyl groups, respectively. In the FT-IR spectra of GO/SiO_2_/PEA, vibration peaks corresponding to C=O, C=C, Si–O–C and N–H are at 1109, 1459, 1628, and 3437 cm^−1^ demonstrated that the chemically attachment of PEA on GO surfaces was done through SiO_2_ linker.

**Fig. 2:**
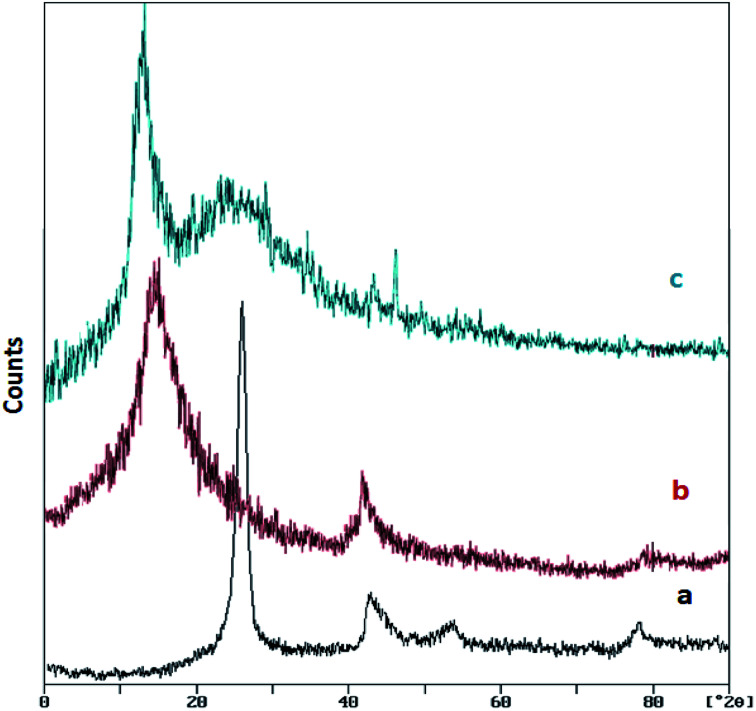
XRD patterns of (a) graphite, (b) GO and (c) GO/SiO_2_/PEA.

Fig. 2 shows the XRD patterns of graphite, GO and GO/SiO_2_/PEA. The XRD pattern of graphite shows a sharp and long peak at about 26.5° (with the intermolecular distance of 0.34 nm). After the Hummer process and graphene oxide formation, the peak is removed and one peak at about 12.7° was appeared (the interlayer distance become 0.7 nm). In the XRD pattern of the GO/SiO_2_/PEA, two signals are observed at 2*θ* = 10.8°, which is due to the structure of the graphene oxide substrate, and the second widespread signal at 2*θ* = 22.8°, indicates that the prepared catalyst has a layered structure contains amine groups.^29^

**Fig. 3:**
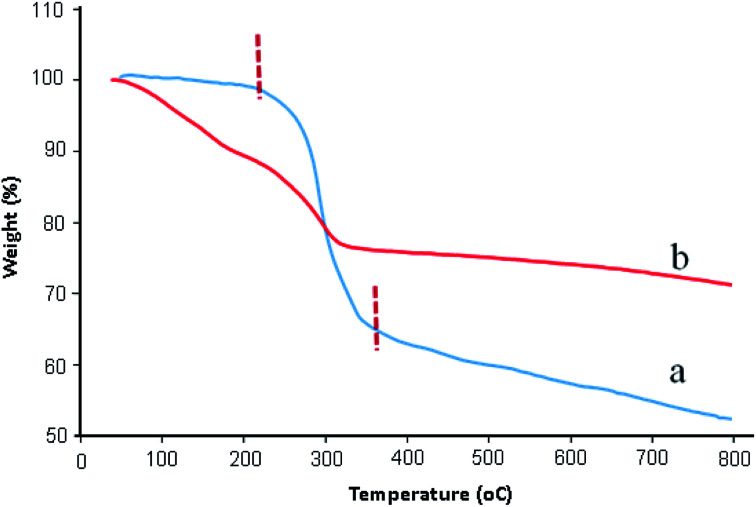
TGA curve of: (a) GO and (b) GO/SiO_2_/PEA.

TGA analysis was used to determine the stability of catalyst and percentage of functional groups attached on graphene oxide surfaces. Fig. 3 exhibited the TGA thermograms of GO and GO/SiO_2_/PEA. As can be seen in Fig. 3a, TGA curve of GO is stable to 220 °C. A major weight loss (about 35%) between 220 °C to 350 °C is due to the decomposition of water and the labile oxygen-containing functional groups on the GO.^30^ As shown in TGA curve of GO/SiO_2_/PEA (Fig. 3b), the weight loss about 5% between 20–120 °C is assigned to removal of physically adsorbed water. The second weight loss (about 20%) occur at the temperatures ranging from 120 to 330 °C, and it is attributed to the removal of chemically grafting of SiO_2_/PEA on to the GO surfaces (Fig. 3b).^31^

**Fig. 4:**
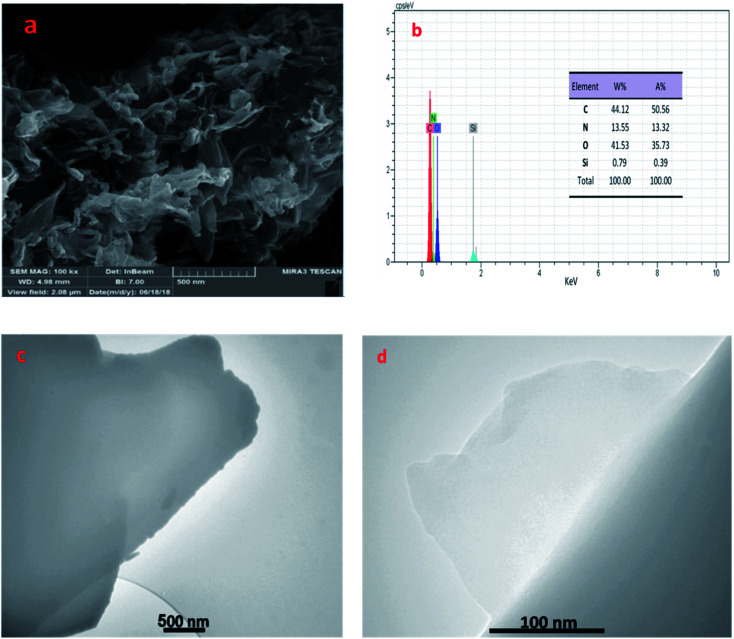
FE-SEM (a), EDX (b) and TEM images (c and d) of GO/SiO_2_/PEA.

In addition, the morphology of prepared catalyst was investigated by the FE-SEM and TEM techniques. As can be seen, the layered, wrinkled and folded structure of catalyst (in Fig. 4a, c and d) proved that SiO_2_/PEA chemically attached on the GO surfaces.^32,33^ Also, EDX was used to prove the composition of GO/SiO_2_/PEA. Results show the presence of nitrogen, silicon, carbon and oxygen in catalyst structure to demonstrate the successfully preparation of GO/SiO_2_/PEA.

**Fig. 5:**
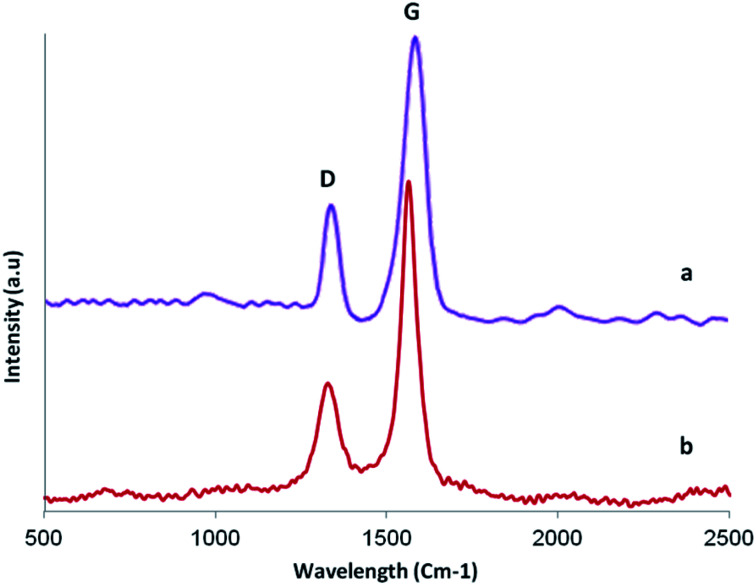
Raman spectra of GO and GO/SiO_2_/PEA.

Raman spectroscopy is a nondestructive technique to characterize the structure of carbon based materials. Fig. 5 shows Raman spectra of GO (curve a) and GO/SiO_2_/PEA (curve b). For carbon materials, the main attributes for a Raman spectra is identified by the D and G bands present around 1345 cm^−1^ and 1571 cm^−1^.^34,35^ The D band, generated by the zone boundary phonons, provides information about the defects and impurities and the G band arises from the doubly degenerate zone centre E_2g_ mode and is present for all carbon based materials.^34,36^Fig. 4 also shows that the position of the G band is shifted from 1571 to 1564 cm^−1^ for GO/SiO_2_/PEA. This is consistent with the restoration of sp^2^ graphitic network.^37^ The intensity ratio *I*_G_/*I*_D_ for both GO and GO/SiO_2_/PEA are found to be around 0.39 indicating similar degrees of disorder which is understandable considering that functionalization of GO occurs by attachment of SiO_2_/PEA predominantly on the defect sites where oxidizing groups were already present and hence it does not generally involve conversion of sp^2^ bonds into sp^3^.^38^

After preparation and characterization of catalyst, to optimize the reaction conditions (catalyst amounts, temperature and solvent), the reaction between ethyl acetoacetate (1 mmol), hydrazine hydrate (1 mmol), isatin (1 mmol) and malononitrile (1 mmol) was used as model reaction. Firstly, optimization of solvent and temperature was studied. Based on the results depicted in Table 1, it is founded that water is the best solvent in terms of yield and time of reaction (at 60 °C) compared to other solvents.

**Table 1:** Optimization of solvent and temperature for the synthesis of spirooxindoles^a^

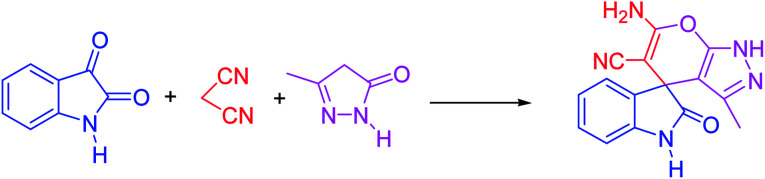				
Entry	Solvent	*T* (°C)	Time (min)	Yield^b^ (%)
1	H_2_O	r.t.	25	80
2	H_2_O	60	18	90
3	C_2_H_5_OH	r.t	25	72
4	C_2_H_5_OH	60	25	83
5	C_2_H_5_OH/H_2_O (1 : 1)	r.t.	25	78
6	C_2_H_5_OH/H_2_O (1 : 1)	60	25	82
7	CH_3_CN	r.t	25	12
8	CH_3_CN	60	25	18

a Ethyl acetoacetate (1 mmol), hydrazine hydrate (1 mmol), isatin (1 mmol) and malononitrile (1 mmol) in the presence of 0.015 g of catalyst.

b Isolated yield.

Additionally, the optimization of catalyst amount and its effect on the reaction time and yield was investigated. The data in Table 2 show that 0.015 g of catalyst cause to the best yield of product and also the further amount of catalyst did not increase the yield of product.

**Table 2:** Optimization of catalyst amount for the synthesis of spirooxindoles^a^

Entry	Catalyst (g)	Time (min)	Yield (%)
1	0.005	30	78
2	0.01	23	84
3	0.015	18	90
4	0.018	18	90
5	0.02	18	90

a (1 mmol) ethyl acetoacetate, (1 mmol) hydrazine hydrate, (1 mmol) isatin, (1 mmol) malononitrile at 60 °C.

After the optimization of reaction conditions, for evaluation of catalyst efficiency, a variety of spirooxindoles were synthesized at optimum conditions. The results and details were summarized in Table 3. As shown in this table, good to excellent yields were achieved after short reaction times in all cases.

**Table 3:** Synthesis of some spirooxindole derivatives using GO/SiO_2_/PEA

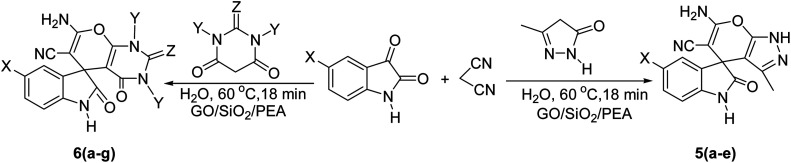					
Entry	Carbonyl compound	Isatin	Product	Yield (%)	Mp (°C)
1	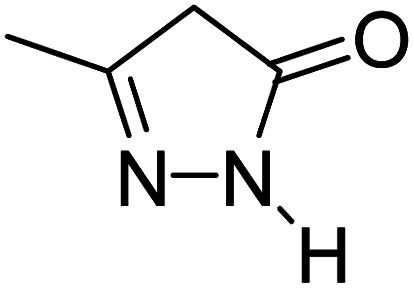	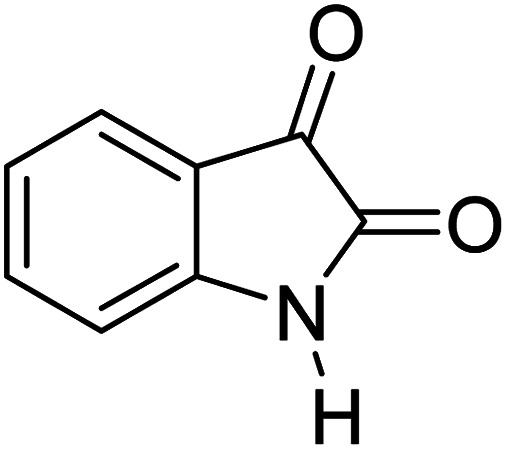	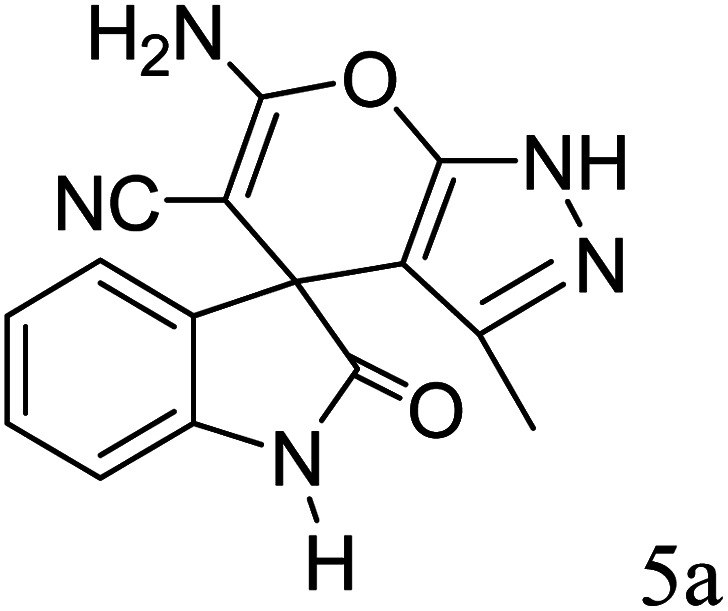	90	232–234 (ref. 28)
2	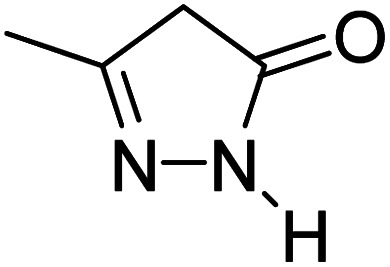	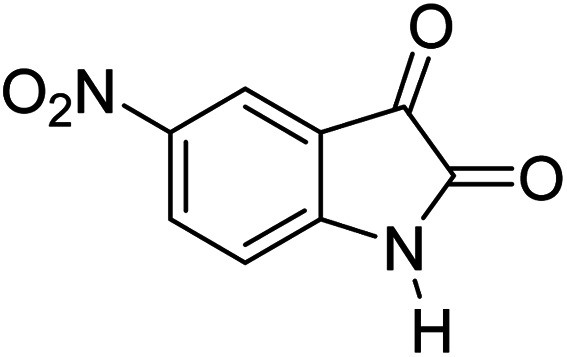	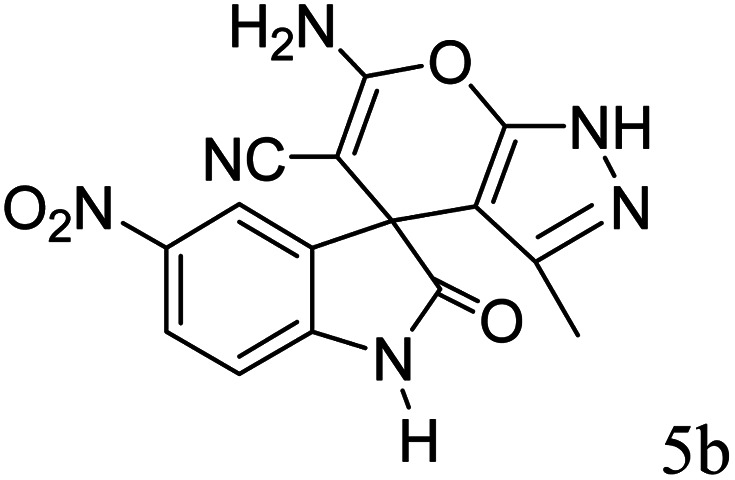	92	226–228 (ref. 28)
3	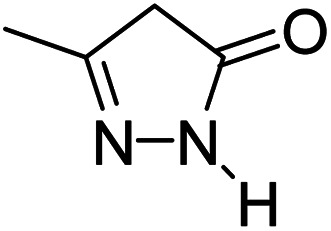	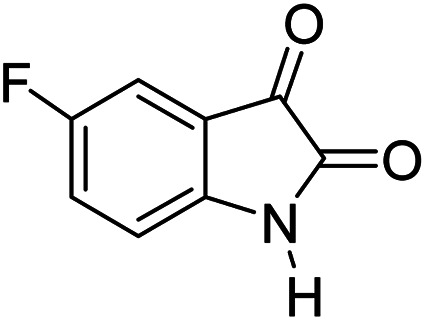	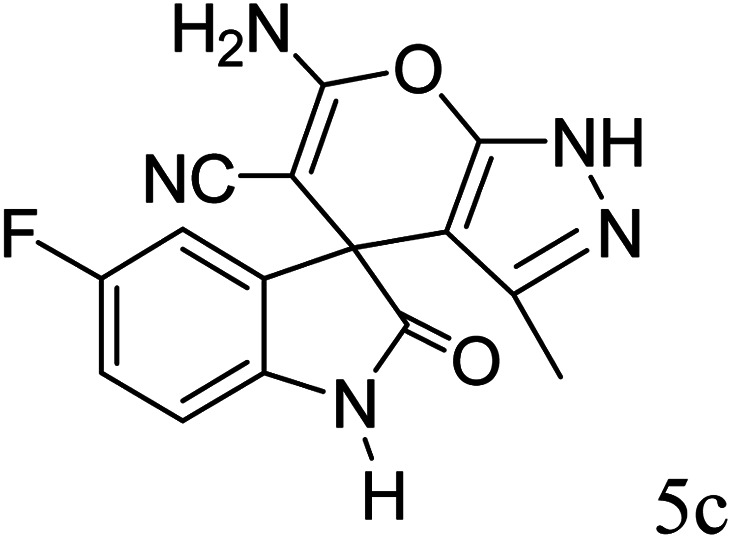	90	262–265 (ref. 27)
4	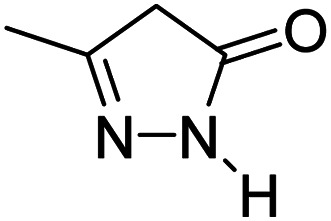	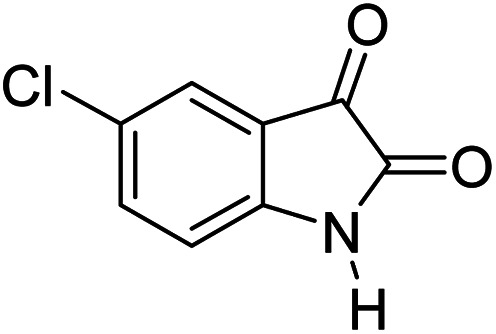	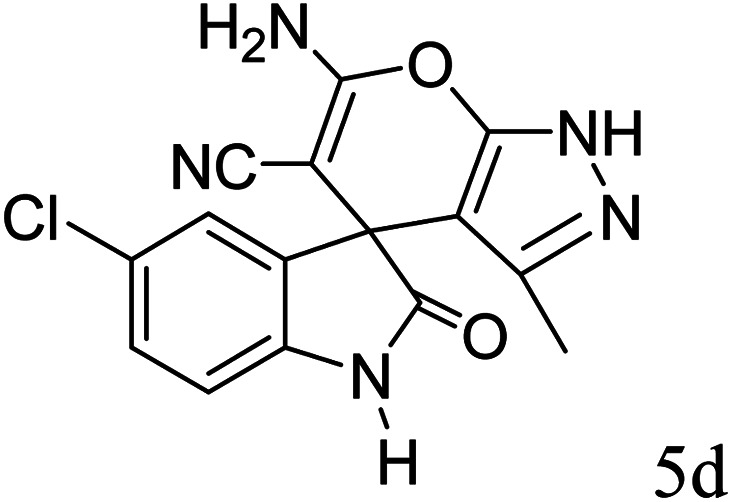	89	232–234 (ref. 27)
5	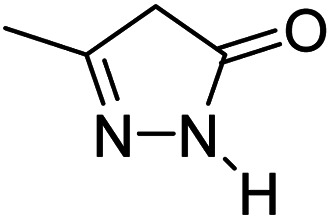	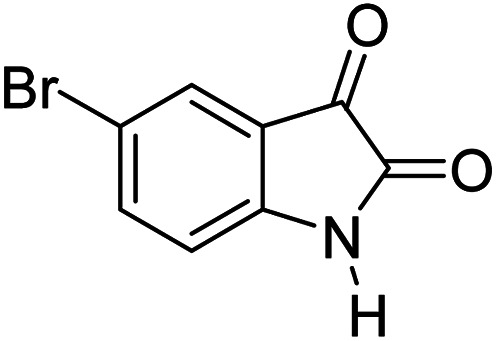	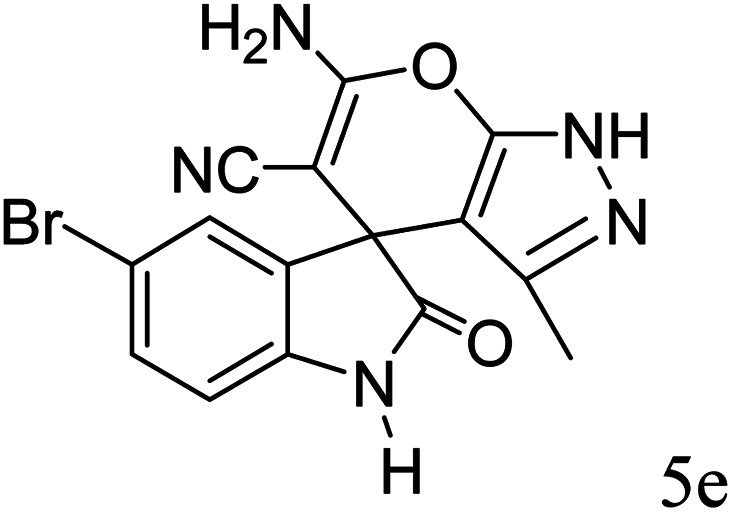	90	248–250 (ref. 27)
6	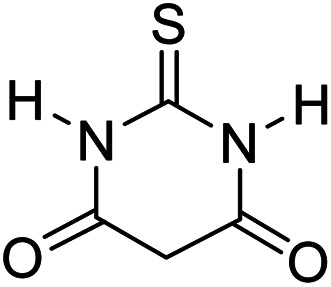	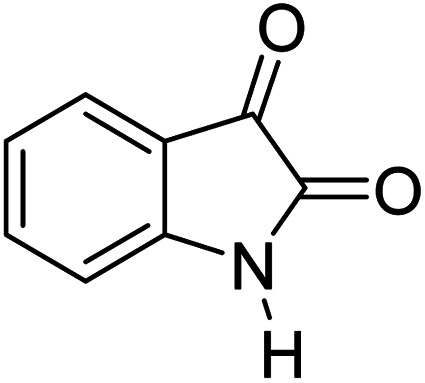	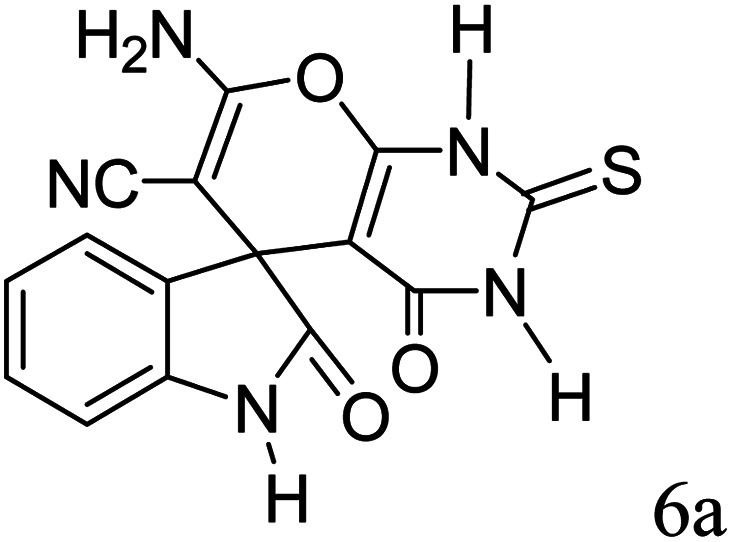	90	281–283 (ref. 39)
7	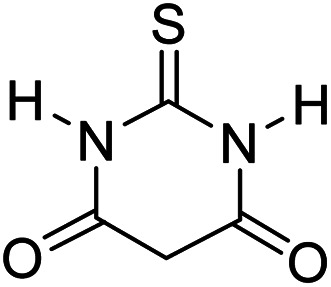	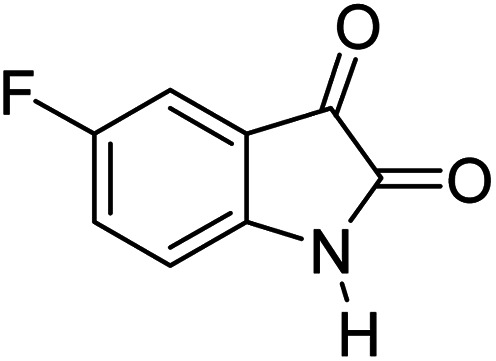	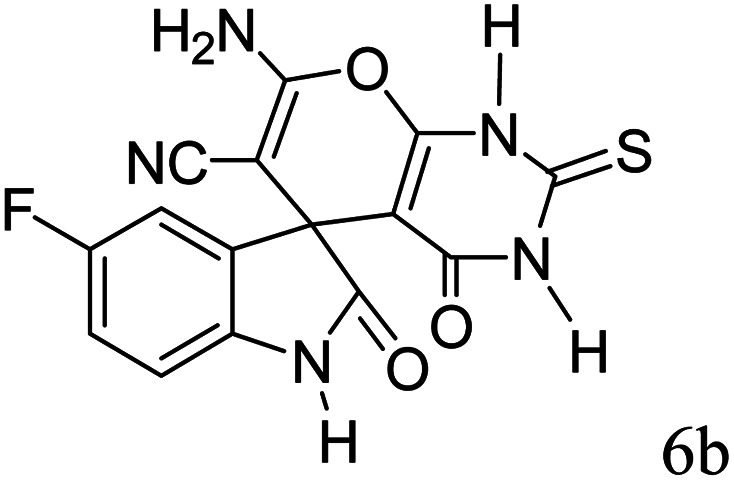	93	299–301 (ref. 39)
8	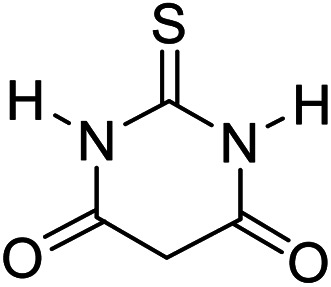	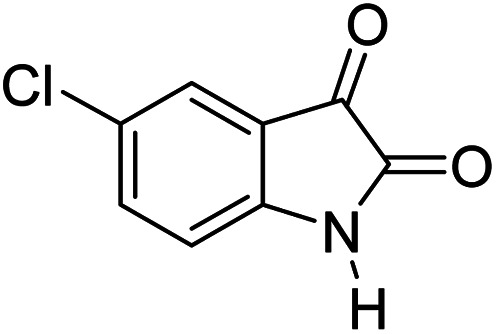	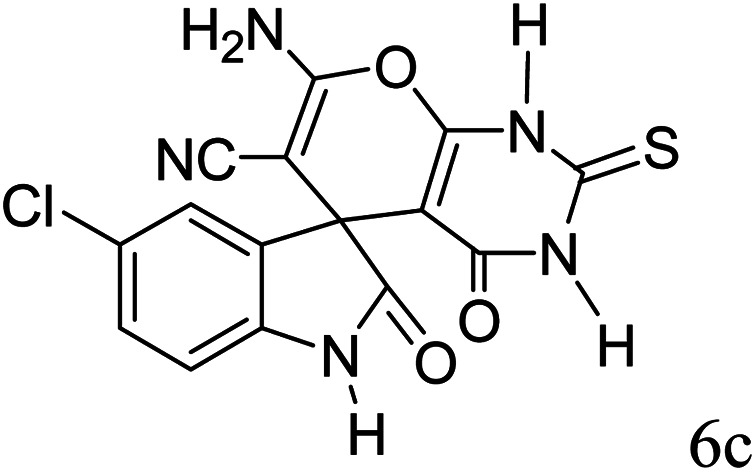	92	289–291 (ref. 39)
9	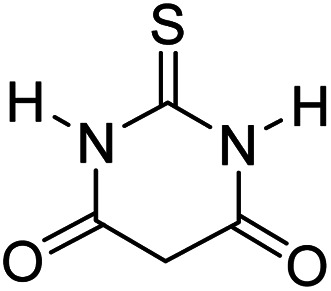	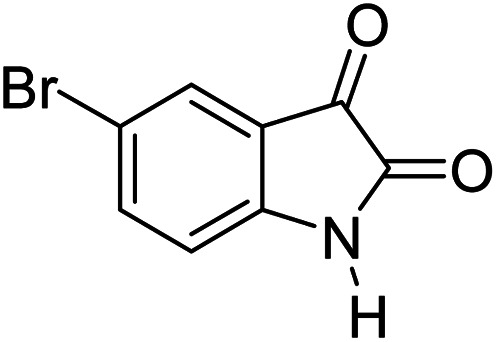	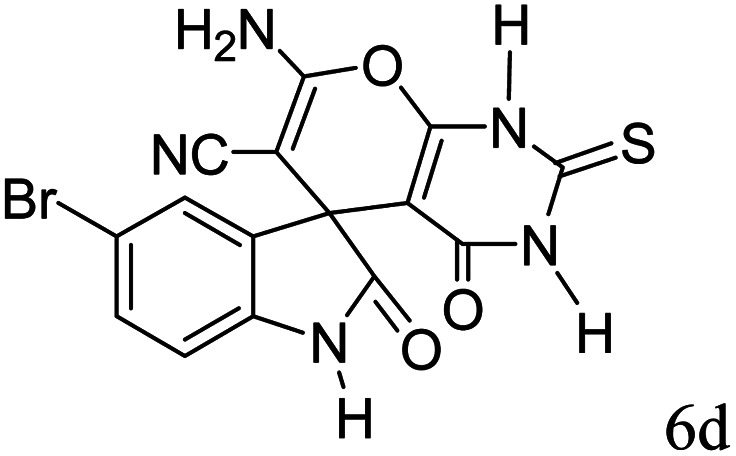	91	238–240 (ref. 39)
10	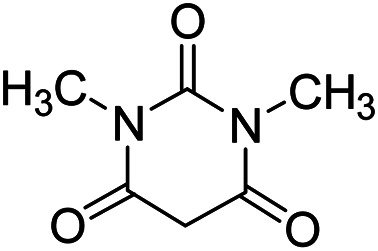	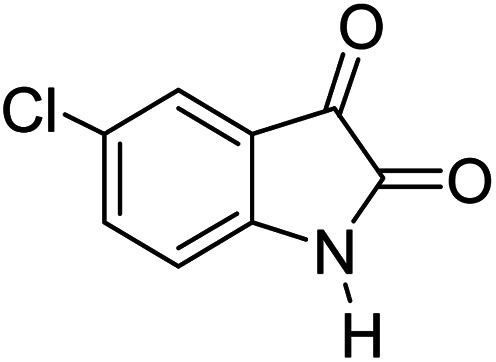	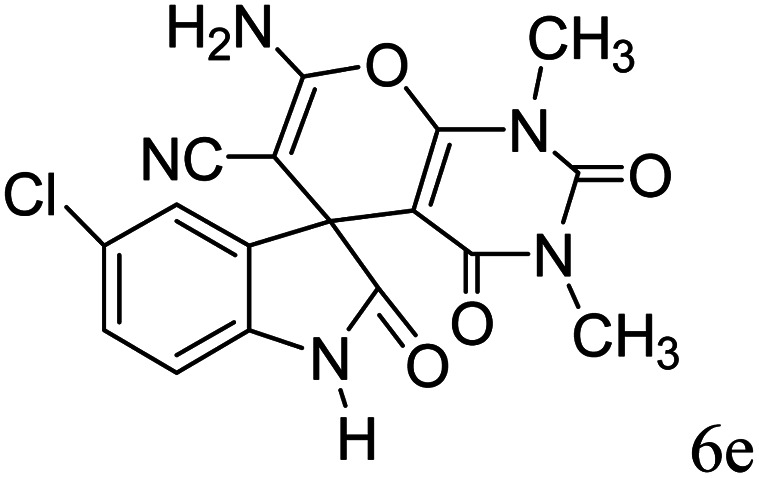	89	248–250 (ref. 33)
11	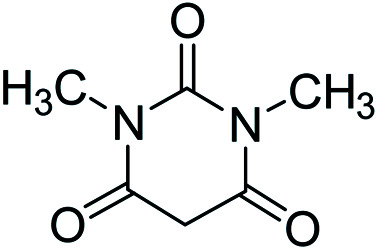	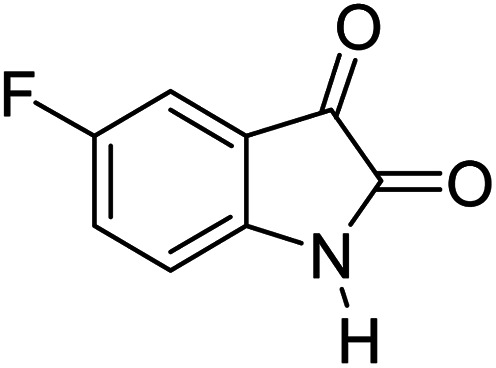	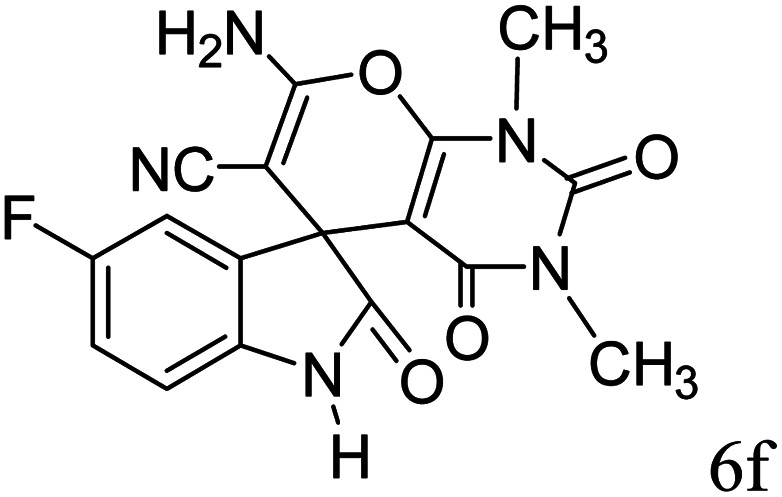	90	276–278
12	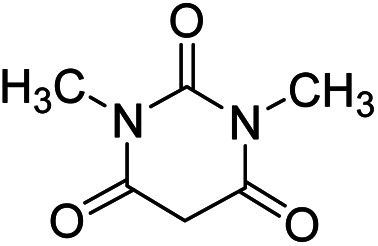	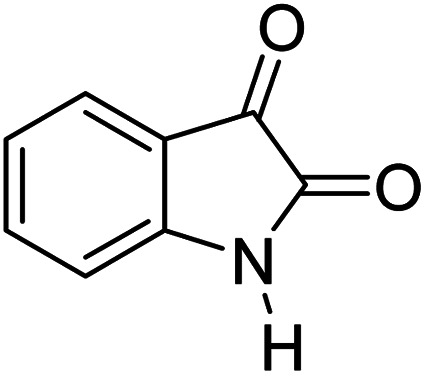	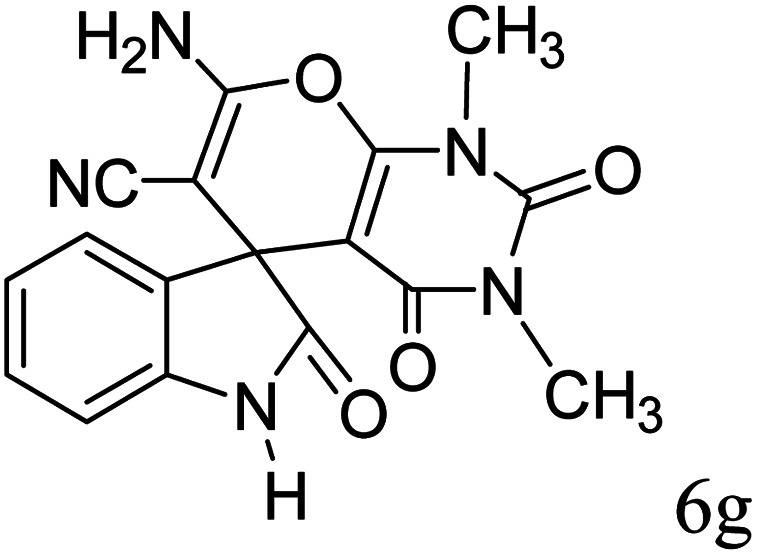	90	299–301 (ref. 20)

**Scheme 3:**
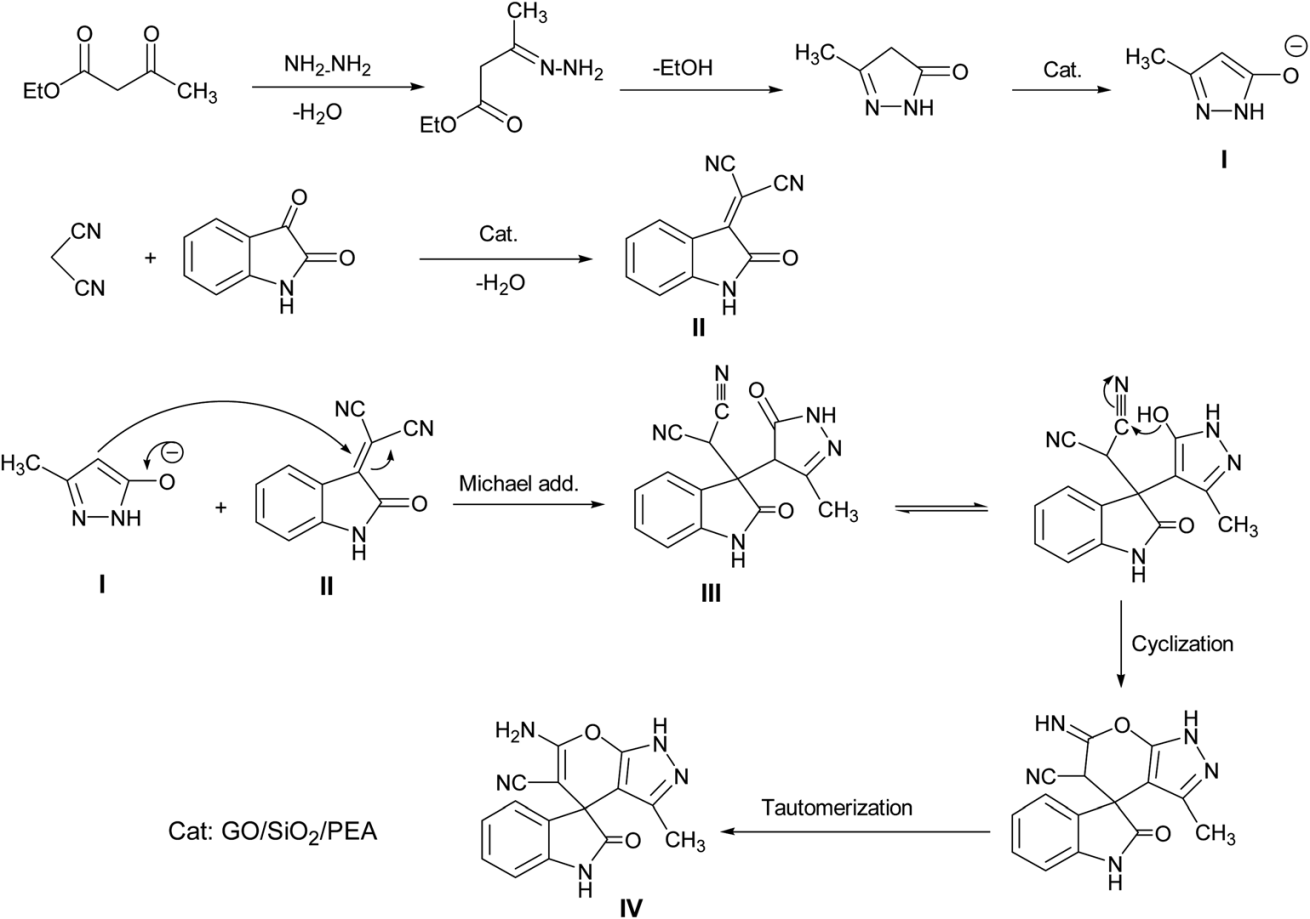
Proposed reaction pathway for the synthesis of spirooxindoles.

A proposed mechanism for the synthesis of spirooxindoles in the presence of GO/SiO_2_/PEA is displayed in Scheme 3. As can be seen, firstly hydrazine hydrate reacts with ethyl acetoacetate to form 3-methyl-2-pyrazoline-5-one (I). On the other hand, Knoevenagel condensation of malononitrile and isatin in the presence of catalyst, lead to formation of intermediate (II). In the next step, Michael addition occurs between intermediate I and II to produce intermediate (III). Finally, the intramolecular cyclization follow by tautomerization of intermediate III resulted the mentioned product.^32,33^

**Fig. 6:**
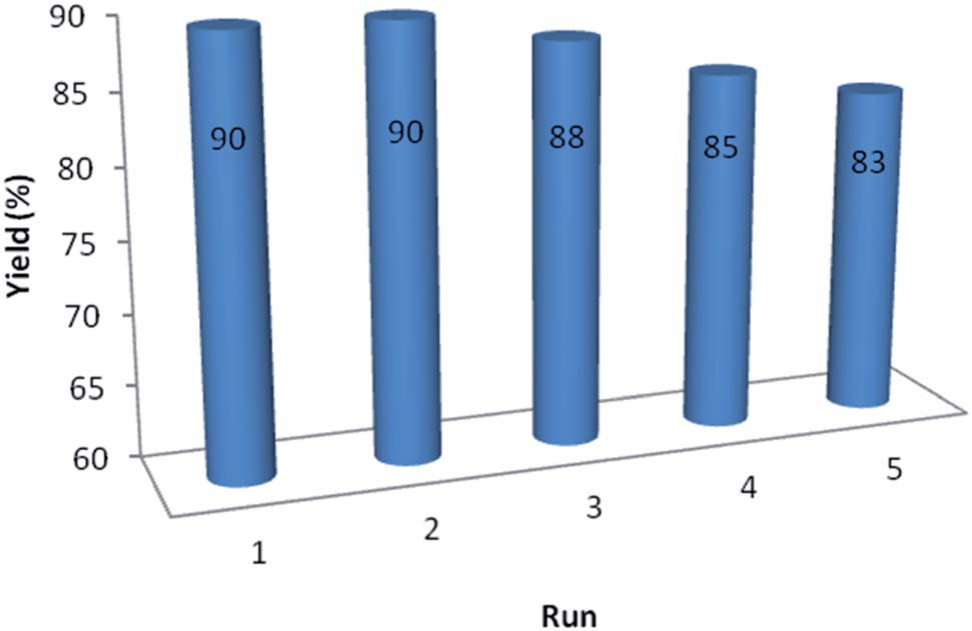
Investigation of catalyst reusability.

In order to study on the performance and recyclability of catalyst, after the completion of sample reaction, catalyst was separated, washed with ethanol and water and dried for using in other similar reaction. Fig. 6 shows that recycled catalyst could be reused five times without considerable decrease in the yield of product to demonstrate the high efficiency and reusability of GO/SiO_2_/PEA.

Hot filtration test has been done to detect the catalyst nature (homogeneous or heterogeneous). In this way, model reaction was performed under optimized conditions. During the test, catalyst was separated from the reaction mixture by filtration after 9 min (half time of reaction), and the reaction of filtrate was continued for other 9 min. The yield of product (about 40%) shown that after removal of the catalyst, the reaction did not proceed; indicating that no catalytically active species (amino groups) remained in the filtrate and the reaction was performed in the presence of heterogeneous GO/SiO_2_/PEA catalyst.

The efficiency of GO/SiO_2_/PEA was compared with some of the other catalysts applied for the synthesis of two kinds of spirooxindole derivatives (5a and 6a). As shown in Table 4, when the reaction was performed in the presence of GO/SiO_2_/PEA in water, the reaction time was shorter and the product yield was higher than others. Green reaction conditions contain to reusable and efficient catalyst, short reaction time, low temperature as well as easy and clean workup, make this method as a green and eco-friendly protocol.

**Table 4:** Comparison of the performance of synthesized catalysts^a^

Entry	Catalyst	Time	Yield (%)	Reference
1	Piperidin	5 h	89	13
2	Et_3_N	5 h	80	14
3	Na_2_CaP_2_O_7_	50 min	78	16
4	l-Proline	30 min	92	17
5	Carbon–SO_3_H	30 min	90	40
6	Meglumine	35 min	92	18
7	THAM^b^ in ethanol, r.t.	4 h	84	41
8	SBA-Pr-NH_2_, r.t., solvent-free	15 min	80	42
9	Chitosan, r.t., (bmim)OH	210 min	90	43
10	EDDF–PEG_60_, r.t.	5 h	87	44
11	Ionic liquid/K_2_CO_3_ in water	1 h	90	45
12	CoFe_2_O_4_@SiO_2_@PUF@Zn(OAc)_2_ in water	45 min	89	46
14	Fe_2_O_3_@SiO_2_@vitB_1_Np in water for sonication	10 min	85	47
15	In water at 6 °C	30 min	83	48
16	γ-Fe_2_O_3_@HAp-Si(CH_2_)_3_SO_3_H in water at 30 °C	20 min	78	49
17	Et_3_N microwave irradiation	2 min	79	50
18	GO/SiO_2_/PEA	18 min	90^c^	This work

a Entry 1–10: reaction conditions for the synthesis of 5a and 11–18 for 6a.

b Tris-hydroxymethylaminomethane.

c The yield of 5a.

## Conclusion

We have established that GO/SiO_2_/PEA is a robust, reusable and heterogeneous solid catalyst for the synthesis of spirooxindoles in water under thermal conditions. This method has several advantages, including excellent yields of products, green reaction solvent, reusability of catalyst and simple work-up process.

## Conflicts of interest

There are no conflicts to declare.

## Supplementary Material

RA-011-D1RA02850B-s001
