# Supplementary material for: Preparation of GO/SiO2/PEA as a new solid base catalyst for the green synthesis of some spirooxindole derivatives
Source: RSC Adv. 2021 Jun 21;11(35):21840–50. doi: 10.1039/d1ra02850b (PMC9034106; doi:10.1039/d1ra02850b)
Supplement: RA-011-D1RA02850B-s001 [file RA-011-D1RA02850B-s001.pdf]

Electronic Supplementary Material (ESI) for RSC Advances.
This journal is © The Royal Society of Chemistry 2021

Preparation of GO/SiO_2/PEA as a new solid base catalyst for the green synthesis of some spirooxindole derivatives

Mahla Toorbaf, Leila Moradi*

Department of Organic Chemistry, Faculty of Chemistry, University of Kashan, P.O. Box 8731753153, Kashan, I. R. Iran

6'-amino-3'-methyl-2-oxo-1'H-spiro[indoline-3,4'-pyrano[2,3-c]pyrazole]-5'-carbonitrile (5a)

M.p. 232-234 °C. FTIR (KBr) υ max: 3337 (NH), 3132 (NH), 2182 (C=N), 1711 (C=O), 1496 (C-H, Ar) Cm^{-1}; ^{1}H NMR (400 MHz, DMSO-d_6) δ_H: 1.51 (3H, s, CH_3), 6.88 (1H, d, *J*=7.0 Hz, ArH), 6.96 (2H, m, ArH), 7.22 (2H, s, NH_2), 7.24 (1H, s, ArH), 10.59 (1H, s, NH), 12.27 (1H, s, NH) ppm; ^{13}C NMR (100 MHz, DMSO-d_6) δ: 12.5, 47.1, 58.5, 111.1, 112.5, 116.2, 122.1, 125.6, 127.7, 140.7, 162.6, 166.9, 175.3 ppm. Anal. Calcd. For $C_{15}H_{11}N_5O_2$: C, 61.43; H, 3.78; N, 23.88 %. Found: C, 61.36; H, 3.75; N, 23.82 %.

6'-amino-3'-methyl-2-oxo-1'H-spiro[5-Nitro-indoline-3,4'-pyrano[2,3-c]pyrazole]-5'-carbonitrile(5b)

M.p. 226-228 °C. FTIR (KBr) υ max: 3419-3337 (NH), 3174 (NH), 2187 (C=N), 1720 (C=O), 1498 (C-H, Ar) Cm^{-1}; ^{1}H NMR (400 MHz, DMSO-d_6) δ_H: 1.57 (3H, s, CH3), 7.12 (1H, d, *J*=7.0 Hz, ArH), 7.41 (2H, s, NH_2), 7.90 (1H, s, ArH), 8.21 (1H, d, *J*=8.0 Hz, ArH), 11.36 (1H, s, NH) , 12.40 (1H, s, NH) ppm; ^{13}C NMR (100 MHz, DMSO-d_6) δ_C: 9.8, 48.3, 55.4, 95.2, 110.8, 119.8, 121.6, 127.3, 134.6, 136.5, 144.5, 148.9, 156.7, 164.1, 178.9 ppm; Anal. Calcd. For $C_{15}H_{10}N_6O_4$: C, 53.25; H, 2.96; N, 24.85 %. Found: C, 53.22; H, 3.12; N, 24.79 %.

6'-amino-3'-methyl-2-oxo-1'H-spiro[5-Fluoro-indoline-3,4'-pyrano[2,3-c]pyrazole]-5' carbonitrile(5c)

M.p. 262-265 °C. FTIR (KBr) υ max: 3409(NH), 3220(NH), 2186(C=N), 1706(C=O), 1490 (C-H, Ar) Cm^{-1}; ^{1}H NMR (400 MHz, DMSO-d_6) δ_H: 1.56 (3H, s, CH_3), 6. 90 (1H, s, ArH), 6. 97 (1H, d, *J*=6.0 Hz, ArH), 7. 07 (1H, d, *J*=7.0 Hz, ArH), 7.28 (2H, s, NH_2), 10.62 (1H, s, NH), 12.31 (1H, s, NH) ppm; ^{13}C NMR (100 MHz, DMSO-d_6) δ_C: 9.4, 48.3, 55.3, 95.4, 111.0, 116.2, 119.1, 134.9, 135.2, 138.3, 147.6, 155.8, 157.8, 163.3, 178.2 ppm; Anal. Calcd. For $C_{15}H_{10}FN_5O_2$: C, 57.82; H, 3.21; N, 22.49 %. Found: C, 57.79; H, 3.23; N, 22.51 %.

6'-amino-3'-methyl-2-oxo-1'H-spiro[5-chloro-indoline-3,4'-pyrano[2,3-c]pyrazole]-5'-carbonitrile (5d)

M.p. 232-234 °C. FTIR (KBr) υ max: 3346 (NH), 3137 (NH), 2182 (C=N), 1714 (C=O), 1487 (C-H, Ar) Cm^{-1}; ^{1}H NMR (400 MHz, DMSO-d_6) δ_H: 1.57 (3H, s, CH_3), 6. 90 (1H, d, *J*=6.0 Hz, ArH), 7. 12 (1H, d, *J*=7.0 Hz, ArH), 7.29 (1H, s, ArH), 7.29 (2H, s, NH_2), 10.74 (1H, s, NH), 12.33 (1H, s, NH) ppm; ^{13}C NMR (100 MHz, DMSO-d6) δ_C: 9.4, 48.1, 55.1, 95.2, 112.0, 119.1, 125.1, 127.1, 129.5, 135.6, 141.3, 155.7, 163.3, 177.9 ppm; Anal. Calcd. For $C_{15}H_{10}ClN_5O_2$: C, 54.88; H, 3.05; N, 21.34 %. Found: C, 54.89; H, 3.09; N, 21.41 %.

6'-amino-3'-methyl-2-oxo-1'H-spiro[5-Bromo-indoline-3,4'-pyrano[2,3-c]pyrazole]-5'-carbonitrile(5e)

M.p. 248-250 °C. FTIR (KBr) υ max: 3346 (NH), 3141 (NH), 2182 (C=N), 1713 (C=O), 1498 (C-H, Ar) Cm^{-1}; ^{1}H NMR (400 MHz, DMSO-d_6) δ_H: 1.56 (3H, s, CH3), 6.86 (1H, d, *J*=6.0 Hz, ArH), 7.22 (1H, s, ArH), 7.30 (2H, s, NH_2), 7.41 (1H, d, *J*=7.0 Hz, ArH), 10.76 (1H, s, NH), 12.34 (1H, s, NH) ppm; ^{13}C NMR (100 MHz, DMSO-d_6) δ_C: 9.4, 48.0, 55.0, 95.2, 112.2, 114.7, 119.5, 128.0, 132.2, 135.2, 135.5, 141.3, 155.6, 163.1, 178.2 ppm; Anal. Calcd. for $C_{15}H_{10}BrN_5O_2$: C, 48.41; H, 2.71; N, 18.82 %. Found: C, 48.44; H, 2.65; N, 18.74 %.

7'-amino-2,4′-dioxo-2′-thioxo-1′,2′,3′,4′-tetrahydrospiro[indoline-3,5'-pyrano[2,3-d]pyrimidine]-6'-carbonitrile (6a)

M.p. 281-283 °C. FTIR (KBr) υ max: 3321 (NH_2), 2197 (C=N), 1679 (C=O amide), 1249 (C=O) Cm^{-1}; ^{1}H NMR (400 MHz, DMSO-d_6) δ_H: 6.77(1H, d, *J*=6.0 Hz, ArH), 6.88 (1H, t, *J*=6.0 Hz ArH), 7.16 (2H, t, *J*=7.0 Hz, ArH), 7.45(2H s, NH_2), 10.56 (1H, s, NH), 12.51 (1H, s, NH) ppm; ^{13}C NMR (100 MHz, DMSO-d_6) δ_c: 45.1, 57.5, 85.2, 112.4, 119.6, 123.7, 124.3, 129.1, 133.7, 142.6, 163.9, 166.1, 167.9, 176.6, 183.2 ppm; Anal. Calcd. for $C_{15}H_9N_5O_2S$: C, 45.79; H, 2.29; N, 17.81 %. Found: C, 45.81; H, 2.27; N, 17.92 %.

7'-amino-5-fluoro-1,1',2,2',3',4'-hexahydro-2,4'-dioxo-2'-thioxospiro[indole-3,5'-pyrano[2,3-d]pyrimidine]-6'-carbonitrile (6b)

M.p. 299-301 °C. FTIR (KBr) υ max: 3367 (NH_2), 3163 (NH), 2199 (C=N), 1684 (C=O amide), 1258 (C=O), 1485(C=C), 1184-1341 (C-O) Cm^{-1}; ^{1}H NMR (400 MHz, DMSO-d_6) δ_H: 6.76(1H, t, *J*=6.0 Hz ArH), 6.95 (1H, t, *J*=6.0 Hz, ArH), 7.18 (1H, d, *J*=7.0 Hz, ArH), 7.44(2H s, NH_2), 10.53 (1H, s, NH), 12.40 (1H, s, NH) ppm; ^{13}C NMR (100 MHz, DMSO-d6) δ_C: 47.1, 57.0, 91.2, 110.0, 112.3, 115.1, 116.8, 134.7, 138.4, 152.9, 157.1, 158.2, 159.2, 174.1, 177.4 ppm; Anal. Calcd. for $C_{15}H_8FN_5O_3S$: C, 50.42; H, 2.24; N, 19.61 %. Found: C, 50.44; H, 2.22; N, 19.59 %.

7'-amino-5-Chloro-1,1',2,2',3',4'-hexahydro-2,4'-dioxo-2'-thioxospiro[indole-3,5'-pyrano[2,3-d]pyrimidine]-6'-carbonitrile (6c)

M.p. 289-291 °C. FTIR (KBr) υ max: 3357 (NH_2), 2197 (C=N), 1681 (C=O amide), 1222 (C=O), 1476 (C=C), 1302-1172 (C-O) Cm^{-1}; ^{1}H NMR (400 MHz, DMSO-d_6) δ_H: 6.77 (1H, d, *J*=6.0 Hz, ArH), 7.19 (1H, d, *J*=7.0 Hz, ArH), 7.36 (1H, s, ArH), 7.45(2H s, NH_2), 10.63 (1H, s, NH) , 12.39 (1H, s, NH) ppm; ^{13}C NMR (100 MHz, DMSO-d_{6},) δ_C: 48.3, 56.8, 91.0, 110.8, 116.9, 124.4, 126.1, 128.6, 135.2, 141.1, 153.3, 158.3, 160.1, 174.3, 177.1 ppm;. Anal. Calcd. for $C_{15}H_8ClN_5O_3S$: C, 48.15; H, 2.14; N, 18.72 %. Found: C, 48.14; H, 2.18; N, 18.81 %.

7'-amino-5-bromo-1,1',2,2',3',4'-hexahydro-2,4'-dioxo-2'-thioxospiro[indole-3,5'-pyrano[2,3-d]pyrimidine]-6'-carbonitrile (6d)

M.p. 238-240 °C. FTIR (KBr) υ max: 3350 (NH_2), 2199 (C=N), 1691 (C=O amide), 1213 (C=O), 1339-1169 (C-O) Cm^{-1}; ^1H NMR (400 MHz, DMSO-d_6) δ_H: 6.74 (1H, d, *J*=6.0 Hz, ArH), 7.32 (1H, t, *J*=7.0 Hz, ArH), 7.50 (1H, S, ArH), 7.50 (2H s, NH_2), 10.68 (1H, s, NH), 12.54 (1H, s, NH) ppm; ^{13}C NMR (100 MHz, DMSO-d6,) δC: 47.3, 57.3, 91.5, 111.7, 114.1, 117.3, 127.5, 131.8, 137.1, 142.5, 153.8, 158.8, 160.0, 174.8, 177.5 ppm; Anal. Calcd. for C15H8BrN5O3S: C, 43.06; H, 1.91; N, 16.74 %. Found: C, 43.11; H, 1.89; N, 16.69 %.

2-amino-6,8-dimethyl-5,7-dioxospiro[5'-chloro-(3'H)-indol-3',4,4(H)-5,6,7,8-tetrahydropyrano(2,3-d)pyrimidine]-(1'H)-2'-one-3-carbonitrile (6e)

M.p. 248-250 °C. FTIR (KBr) υ max: 3407(NH_2), 2197(C=N), 1681(C=O amide), 1245(C=O), 1499(C=C), 1302-1172(C-O) Cm^{-1}; ^1H NMR (400 MHz, DMSO-d_6) δ_H: 3.02 (3H, S, CH_3), 3.35 (3H, S,CH_3), 6.79 (1H, d, *J*=6.0 Hz, ArH), 7.18 (1H, d, *J*=7.0 Hz, ArH), 7.27(1H s, ArH),7.64 (2H, s, NH_2), 10.63 (1H, s, NH) ppm; ^{13}C NMR (100 MHz, DMSO-d_6) δ_c: 28.2, 29.7, 48.1, 56.9, 86.1, 113.5, 117.2, 118.0, 126.4, 130.6, 136.3, 140.6, 151.1, 152.6, 157.9, 165.9, 178.1 ppm; Anal. Calcd. for $C_{17}H_{12}ClN_5O_4$: C, 31.10; H, 3.11; N, 18.13 %. Found: C, 31.12; H, 3.09; N, 18.15 %.

2-amino-6,8-dimethyl-5,7-dioxospiro[5'-Fluoro-(3'H)-indol-3',4,4(H)-5,6,7,8-tetrahydropyrano(2,3-d)pyrimidine]-(1'H)-2'-one-3-carbonitrile (6f)

M.p. 276-278 °C. FTIR (KBr) υ max: 3484(NH_2), 2197(C=N), 1681(C=O amide), 1288(C=O), 1486 (C=C) Cm^{-1}; ^1H NMR (400 MHz, DMSO-d_6) δ_H: 3. 01 (3H, S, CH_3), 3. 46 (3H, S, CH_3), 6.77 (1H, m, ArH), 6.97 (1H, t, *J*=6.0 Hz ArH), 7.07 (1H, s, ArH), 7.62 (2H, s, NH_2), 10.52 (1H, s, NH), 12.50 (1H, S, NH) ppm; ^{13}C NMR (100 MHz, DMSO-d6) δC: 28.2, 30.1, 47.8, 56.9, 85.4, 87.1, 112.3, 117.2, 132.6, 136.9, 137.5, 142.3, 150.1, 153.1, 159.1, 161.0, 178.3 ppm; Anal. Calcd. for $C_{17}H_{12}FN_5O_4$: C, 32.49; H, 3.24; N, 18.95 %. Found: C, 32.46; H, 3.28; N, 18.91 %.

7'-amino-1,1′,2,2′,3′,4′-hexahydro-1′,3'-dimethyl-2,2′,4'-trioxospiro[indole-3,5'-pyrano[2,3-d]pyrimidine]-6'-carbonitrile (6g)

M.p. 299-301°C. FTIR (KBr) υ max: 3428 (NH_2), 3317 (NH), 2924 (CH_2), 2000 (C=N), 1693 (C=O amide), 1652 (C=O) Cm^{-1}; ^1H NMR (400 MHz, DMSO-d_6) δ_H: 3.01 (3H, s, CH_3), 3.71 (3H, s, CH_3), 6.95 (1H, d, *J*=6.0 Hz ArH). 7.28 (2H, t, *J*=7.0 Hz, ArH), 7.43 (1H, t, *J*=7.0 Hz, ArH), 7.13 (2H, s, NH_2) ppm; ^{13}C NMR (100 MHz, DMSO-d6) δC: 27.4, 29.6, 48.0, 57.5, 87.3, 109.6, 117.0, 121.8, 123.9, 128.4, 133.5, 142.2, 150.0, 152.1, 158.3, 159.2, 177.5 ppm; Anal. Calcd. for $C_{17}H_{13}N_5O_4$: C, 34.16; H, 3.70; N, 19.94 %. Found: C, 43.14; H, 3.73; N, 19.97 %.

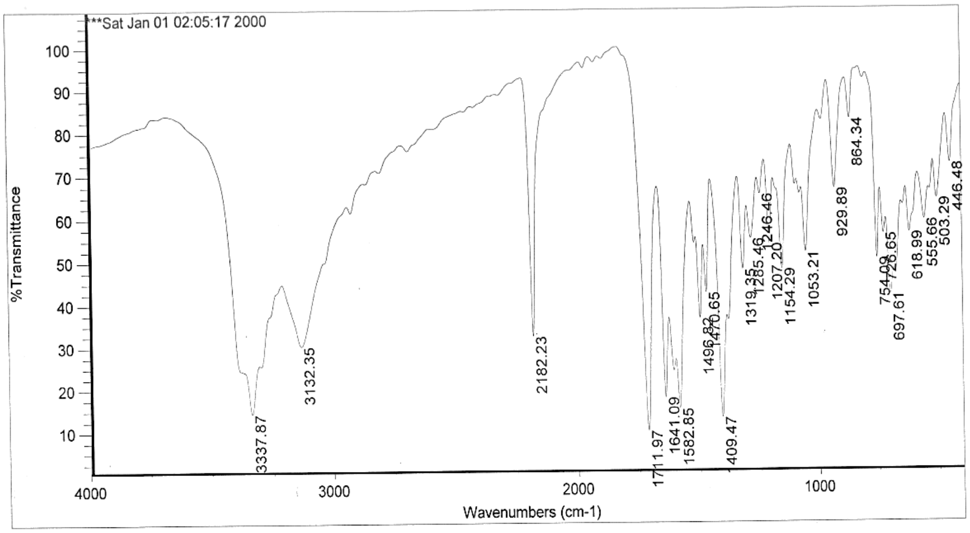

FTIR of 5a

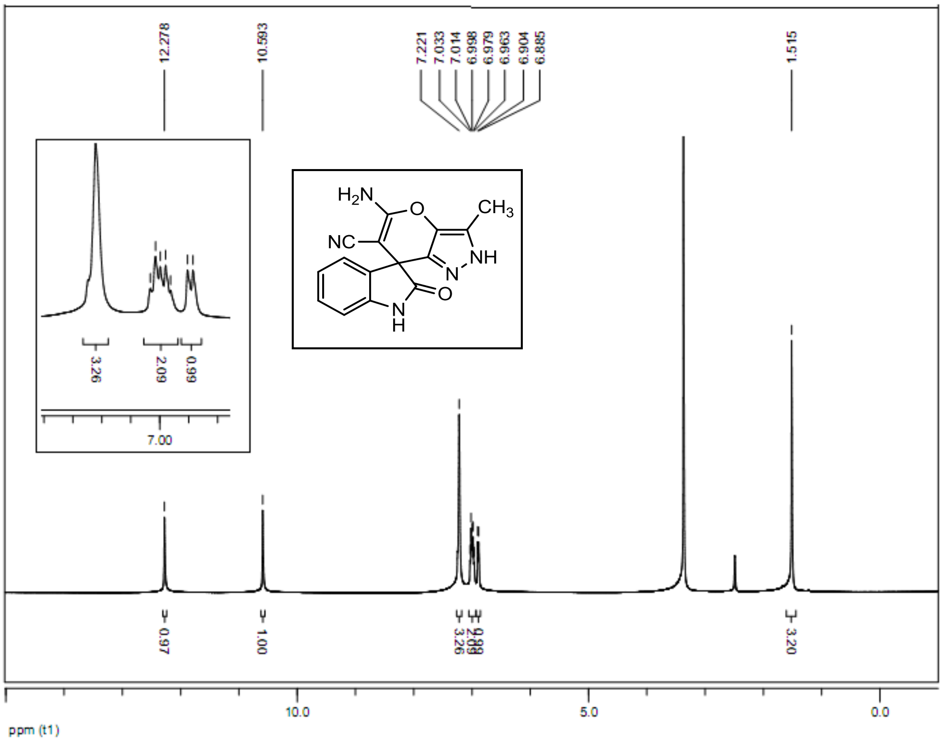

^{1}HNMR of 5a

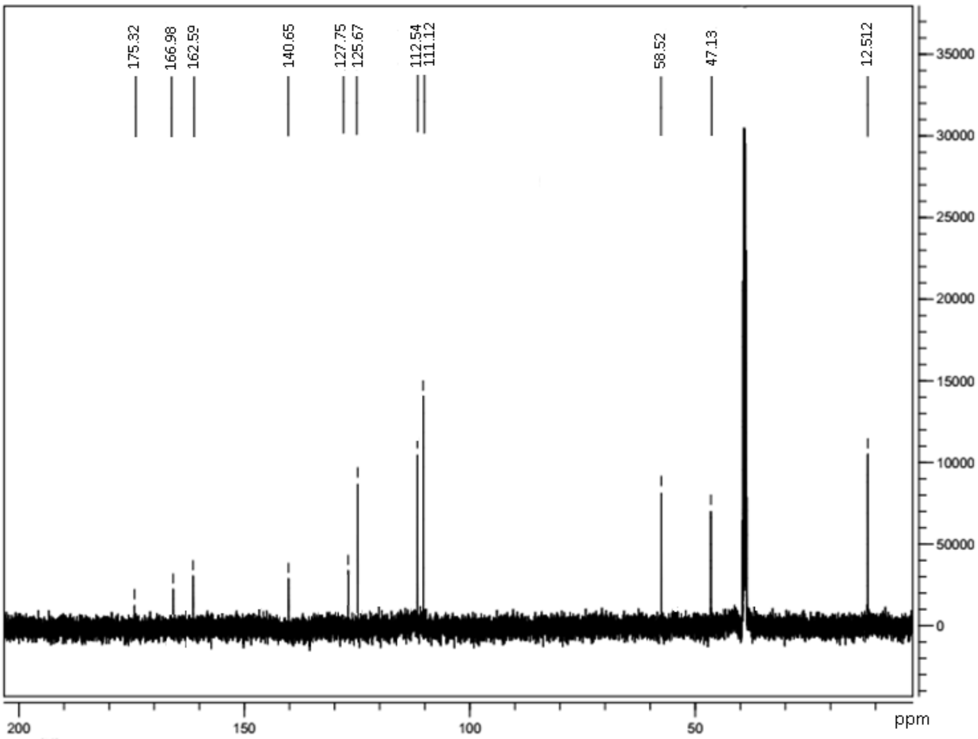

^{13}C NMR of 5a

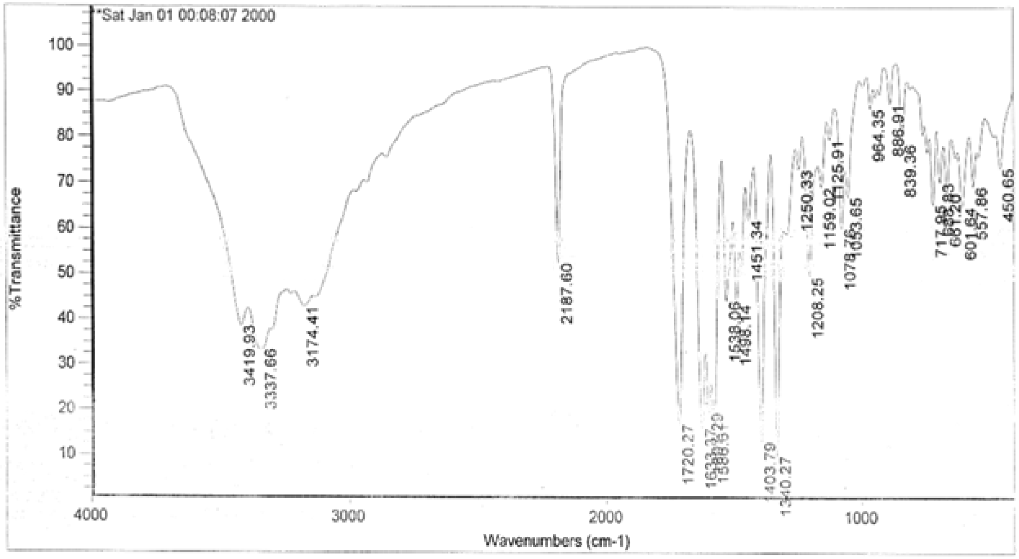

FTIR of 5b

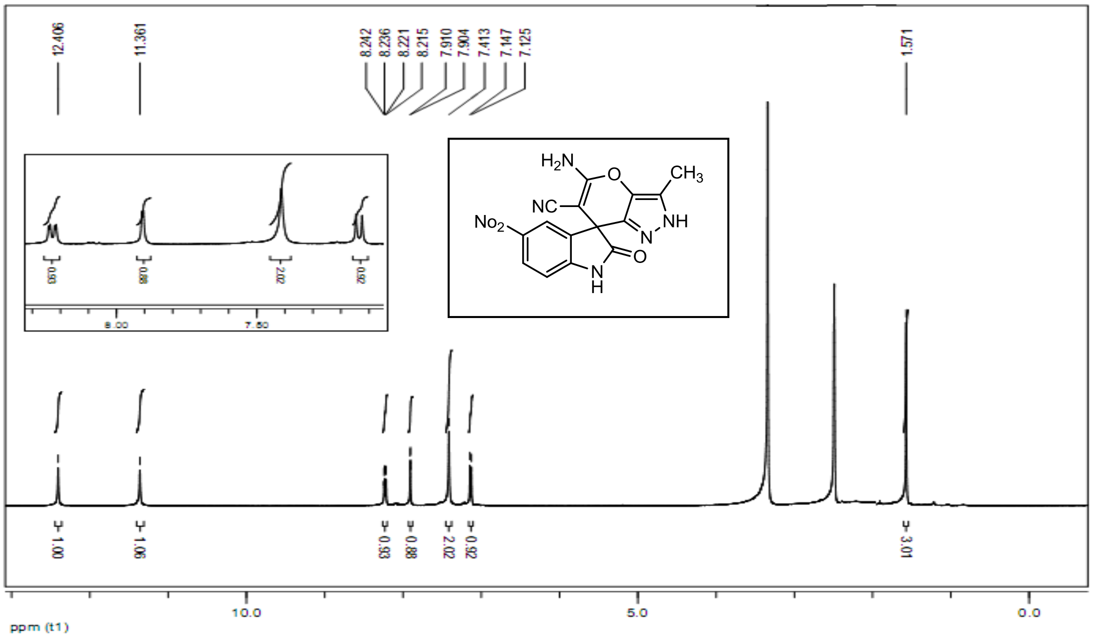

^{1}HNMR of 5b

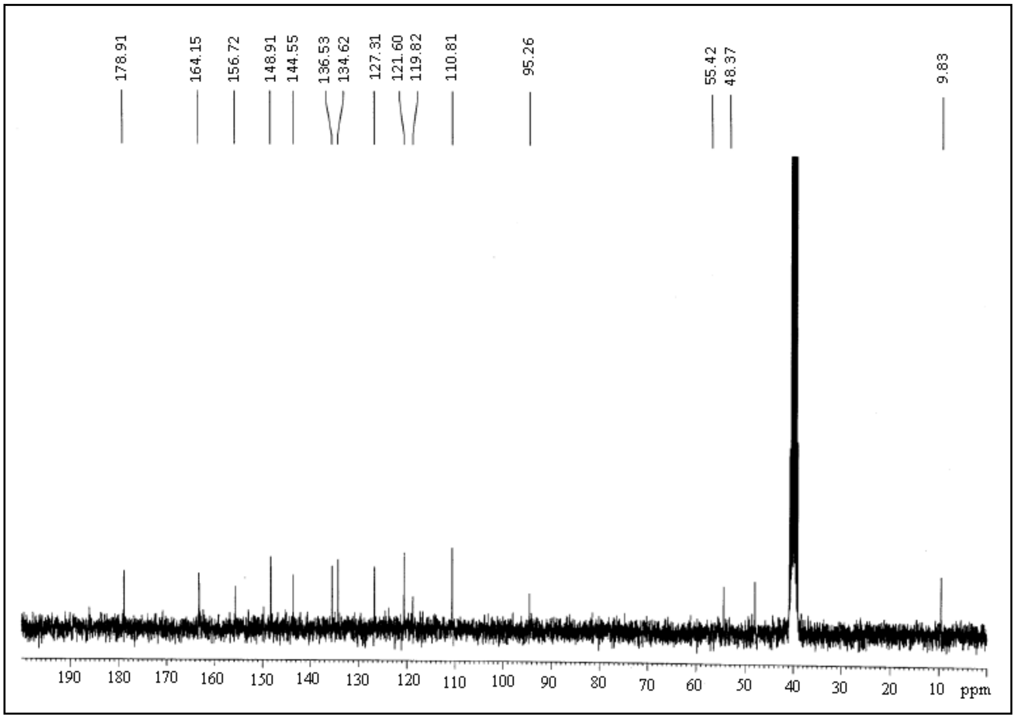

^{13}C NMR of 5b

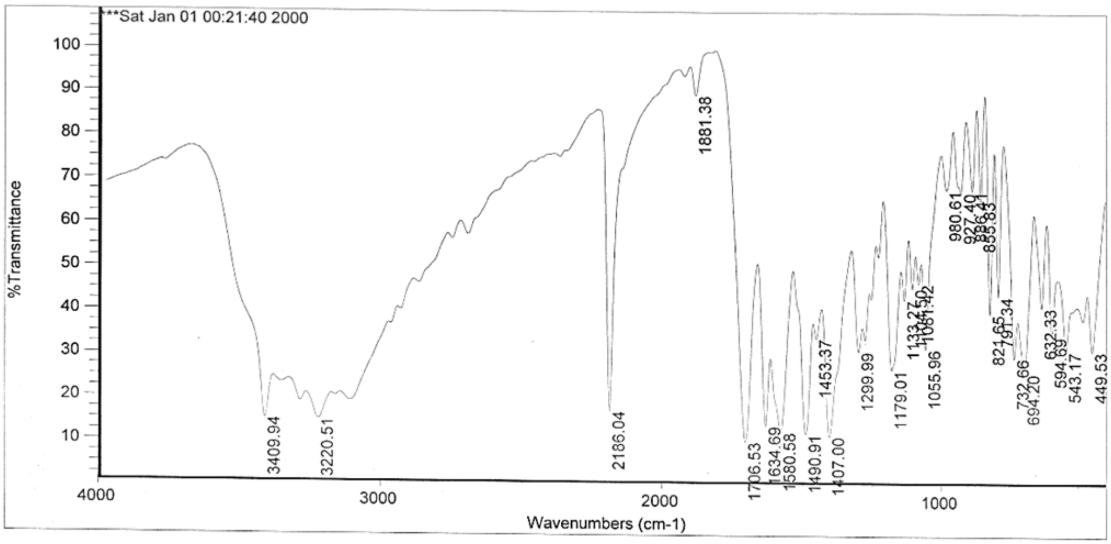

FTIR of 5c

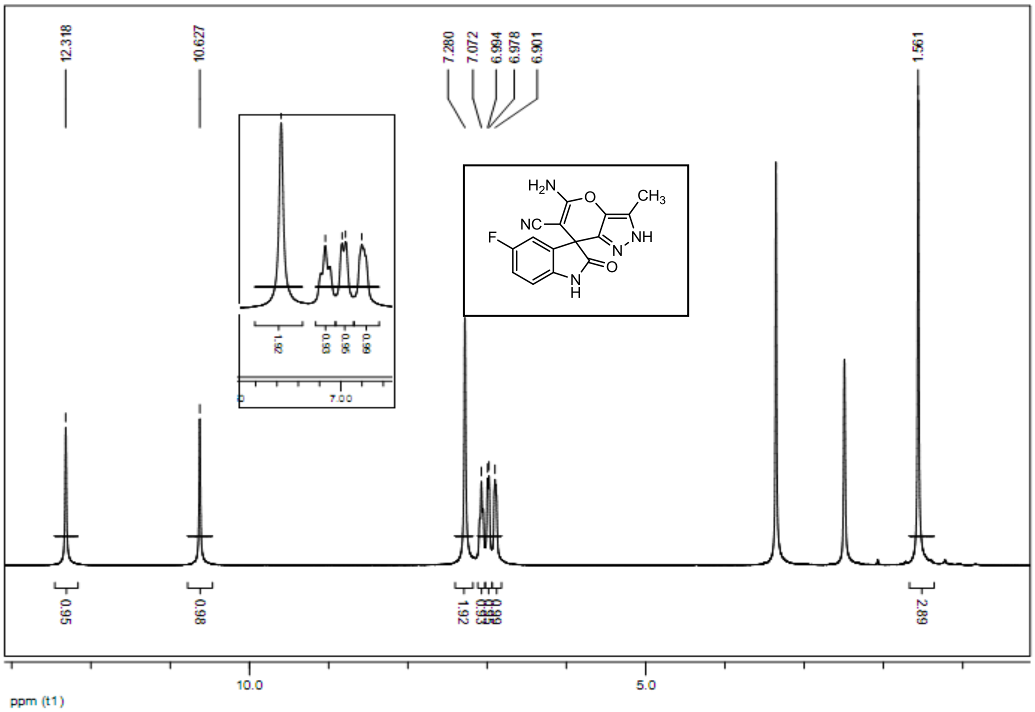

^{1}HNMR of 5c

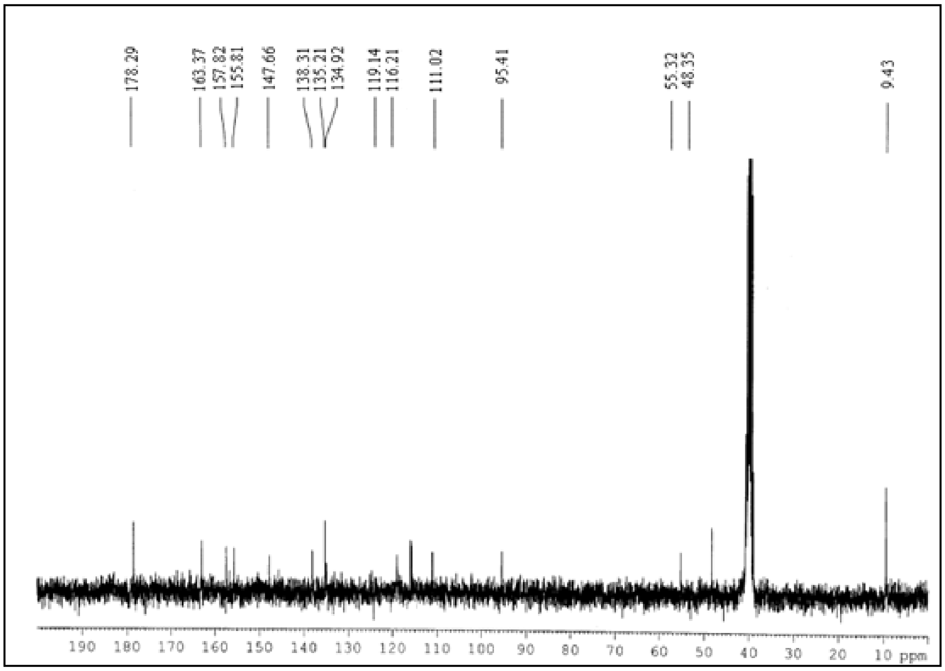

^{13}C NMR of 5c

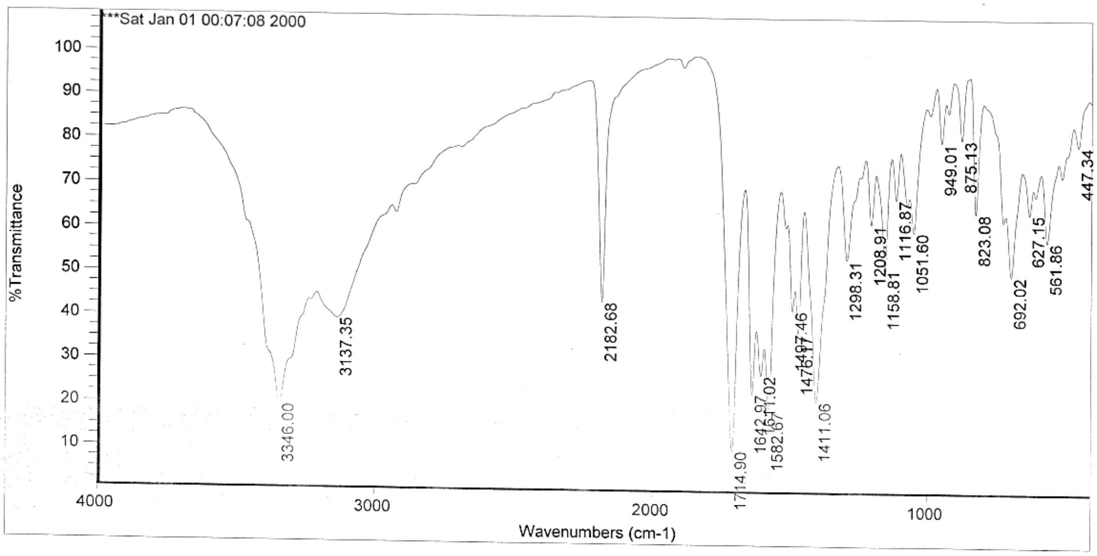

FTIR of 5d

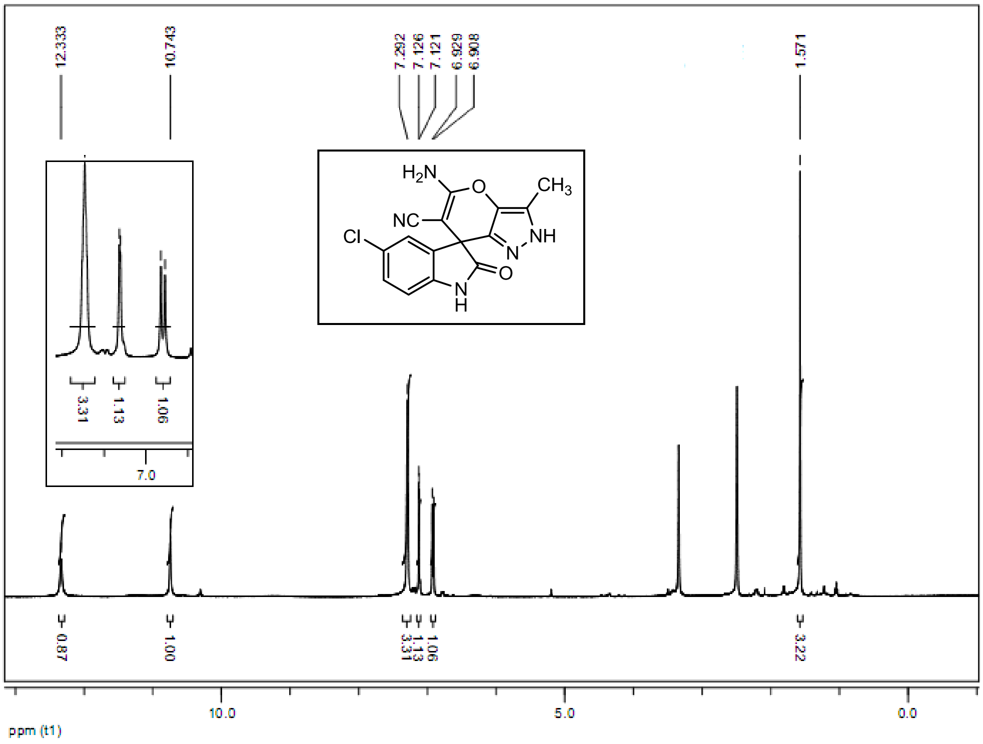

^{1}HNMR of 5d

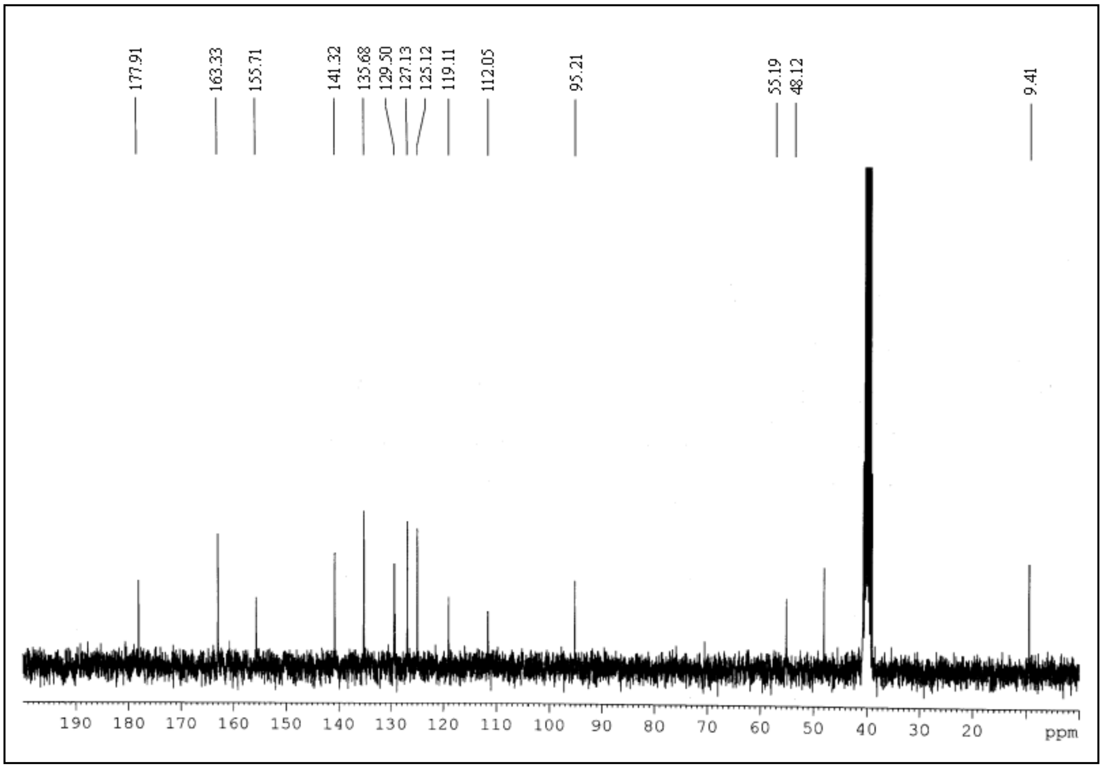

^{13}C NMR of 5d

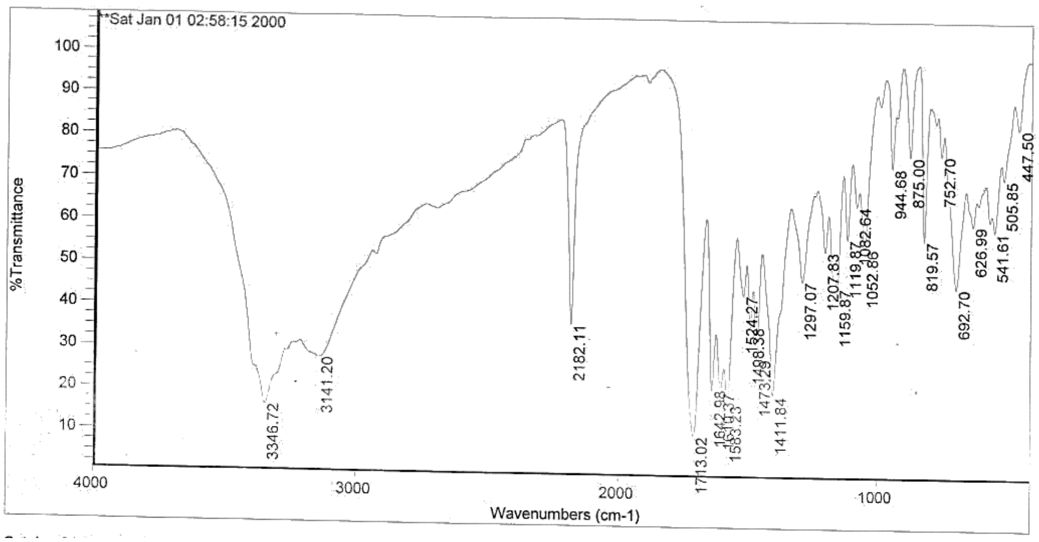

FTIR of 5e

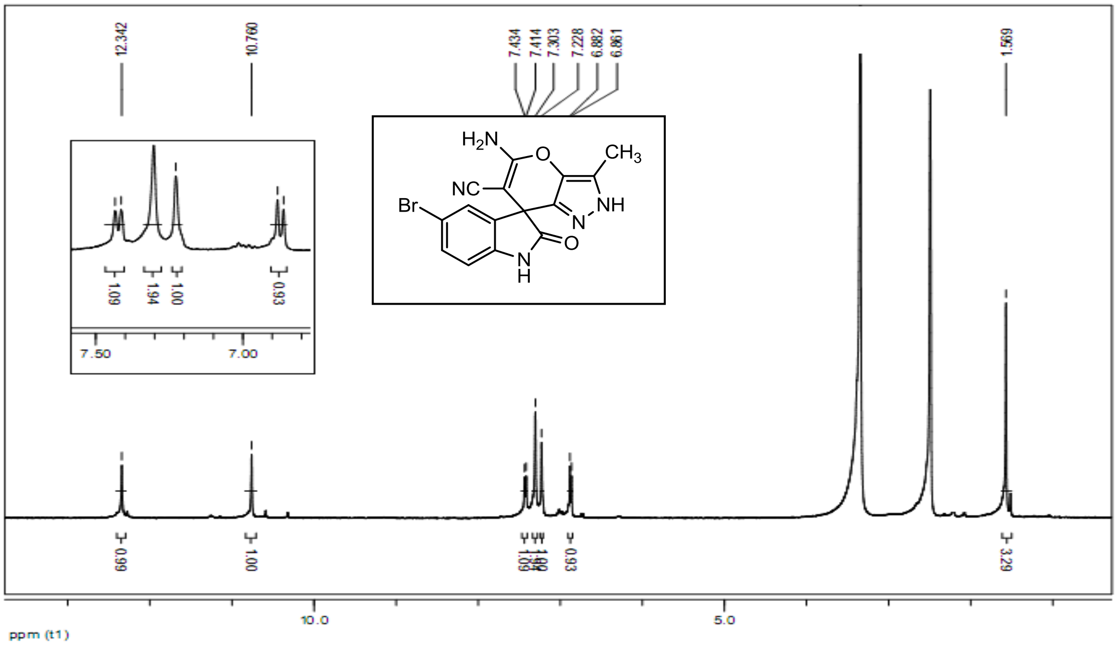

^{1}HNMR of 5e

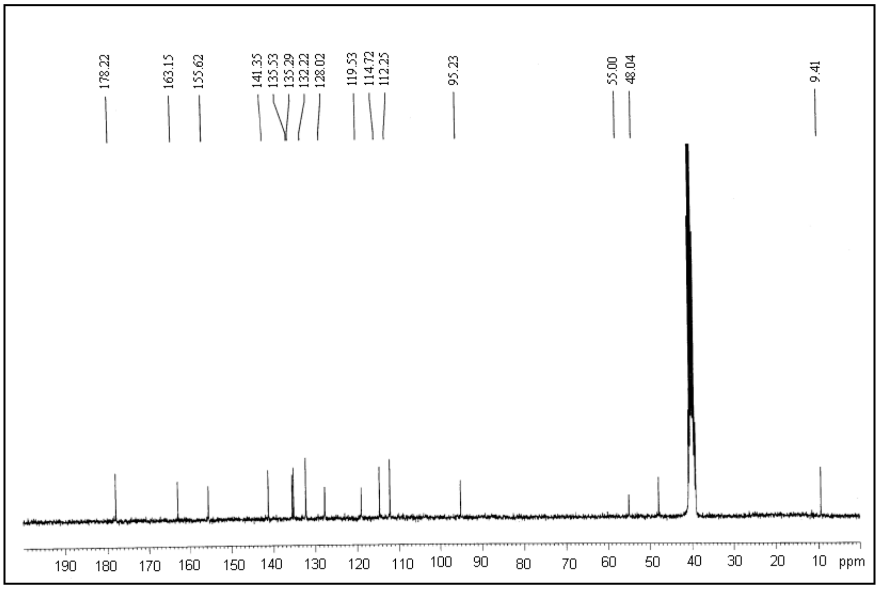

^{13}C NMR of 5e

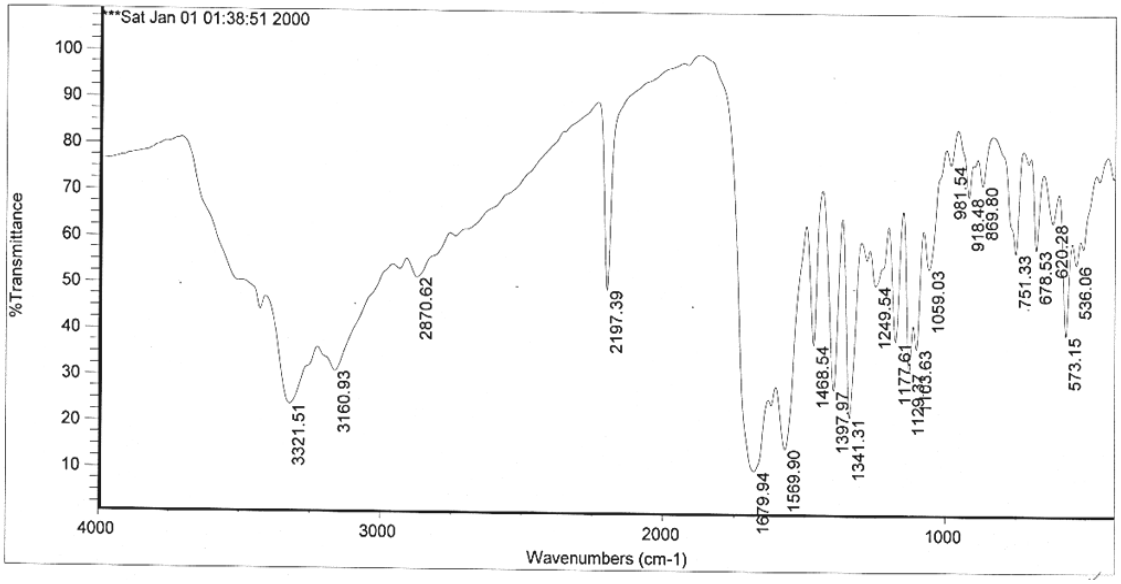

FTIR 6a

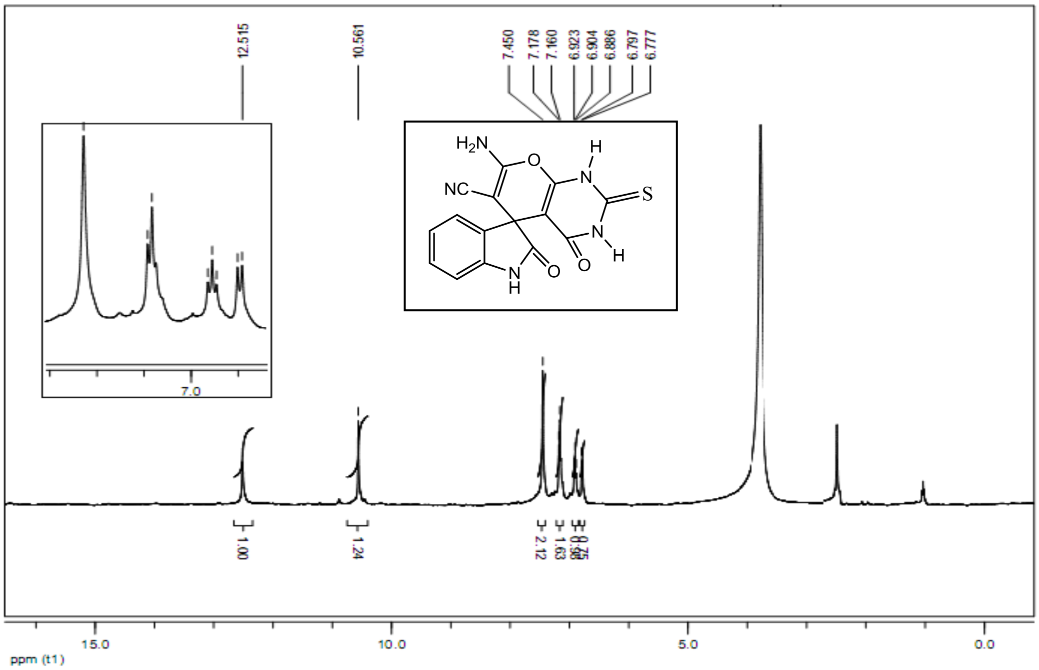

^{1}HNMR of 6a

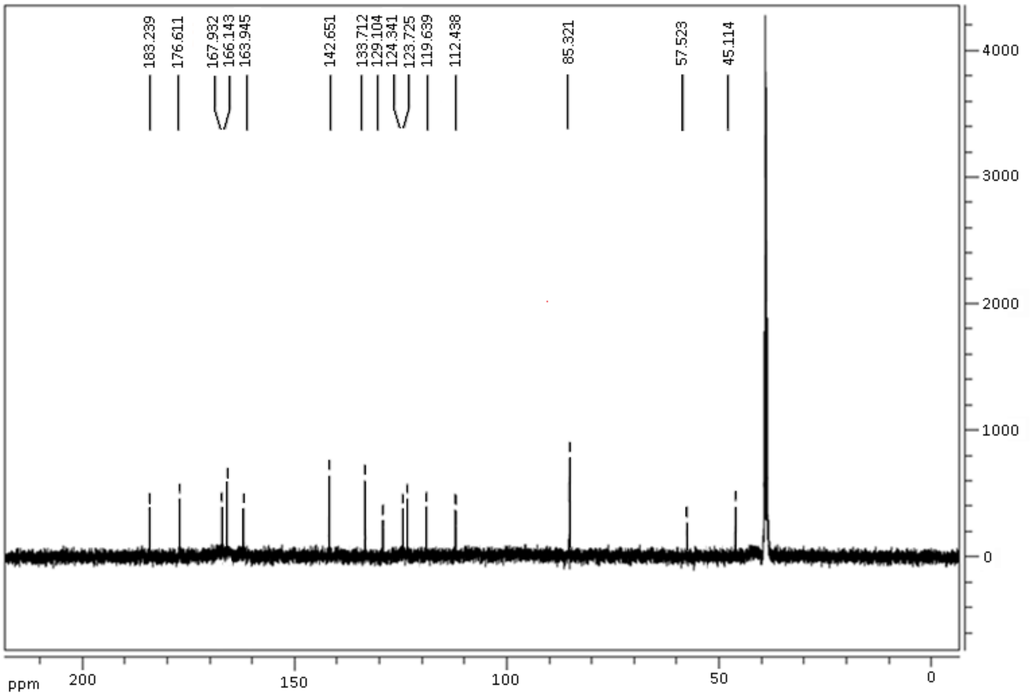

^{13}C NMR of 6a

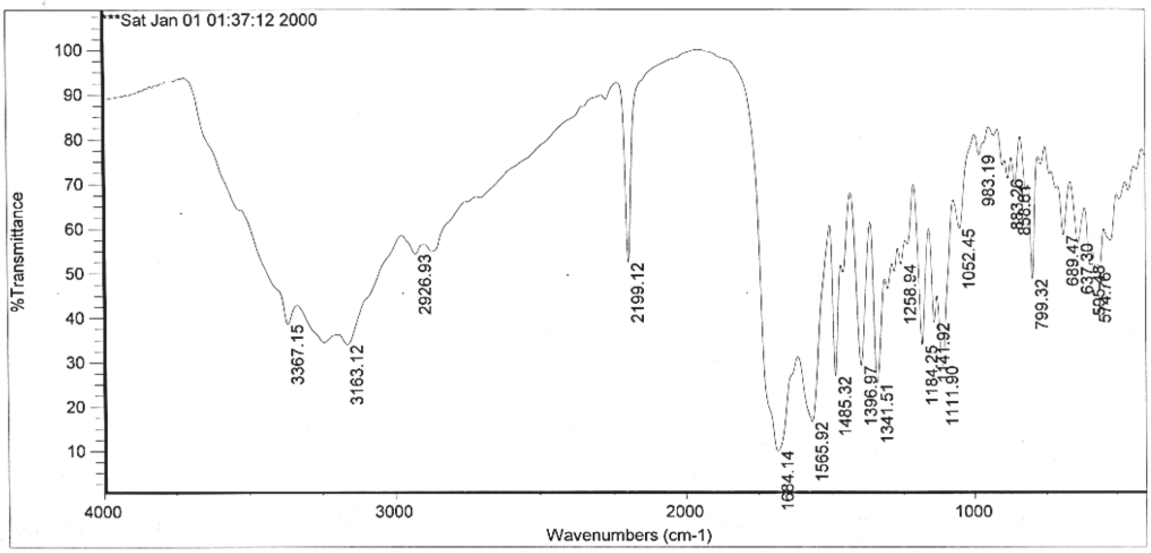

FTIR of 6b

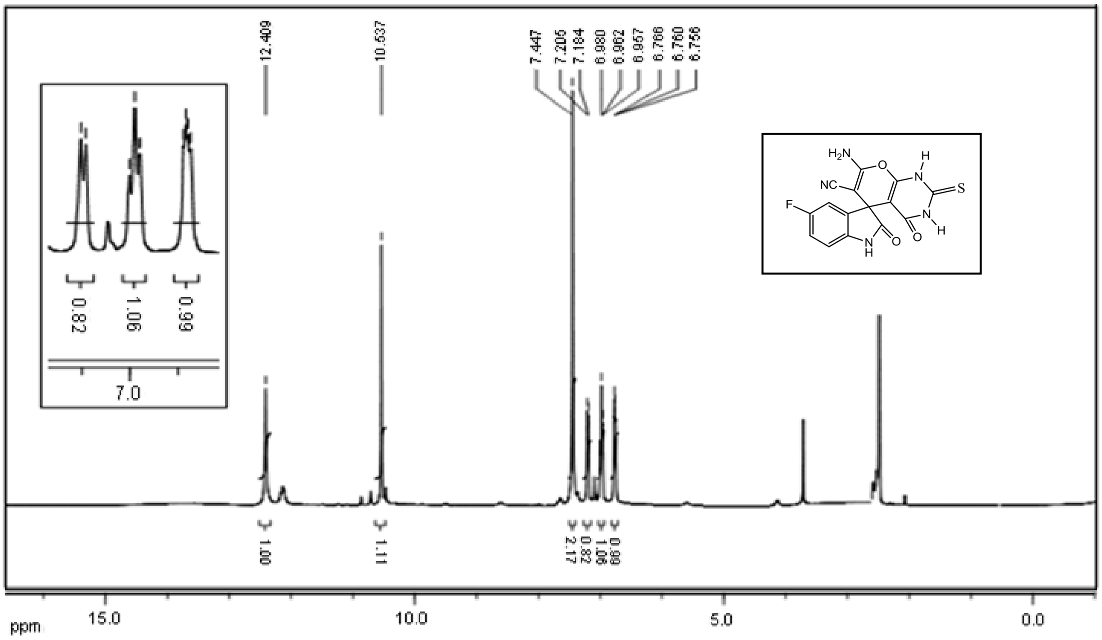

^{1}HNMR of 6b

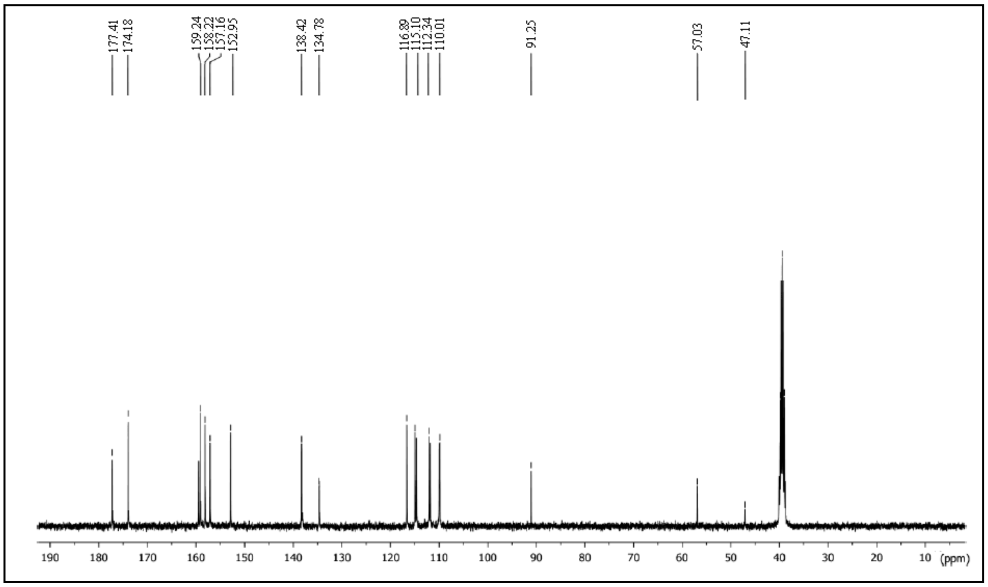

^{13}C NMR of 6b

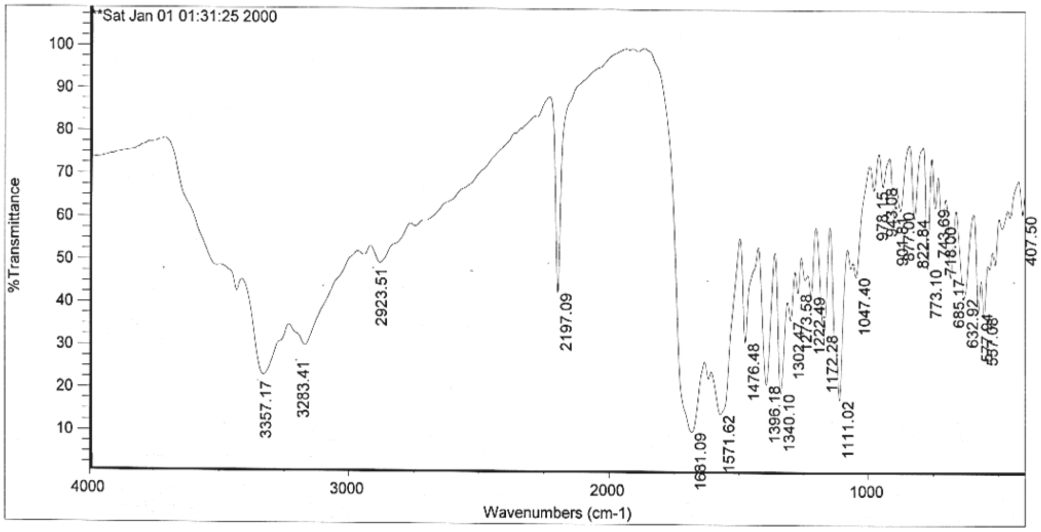

FTIR of 6c

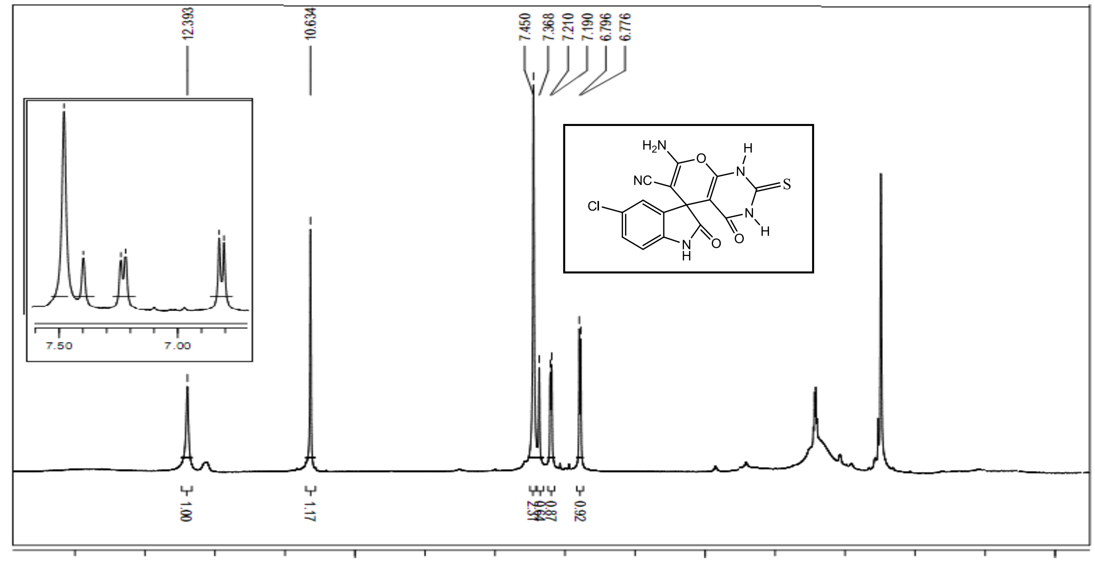

^{1}HNMR of 6c

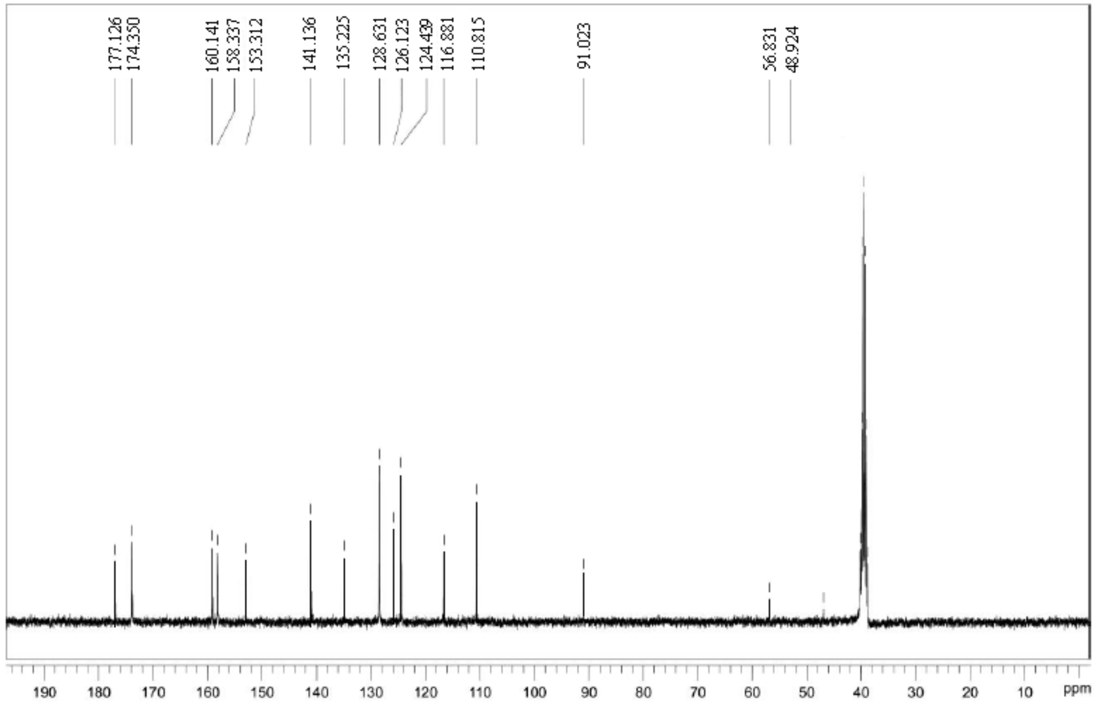

^{13}C NMR of 6c

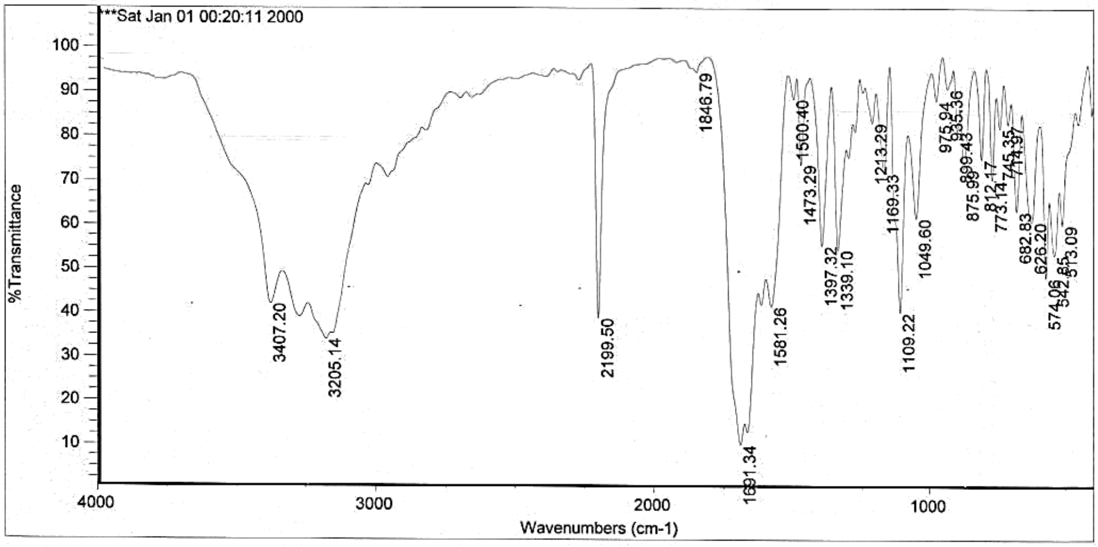

FTIR of 6d

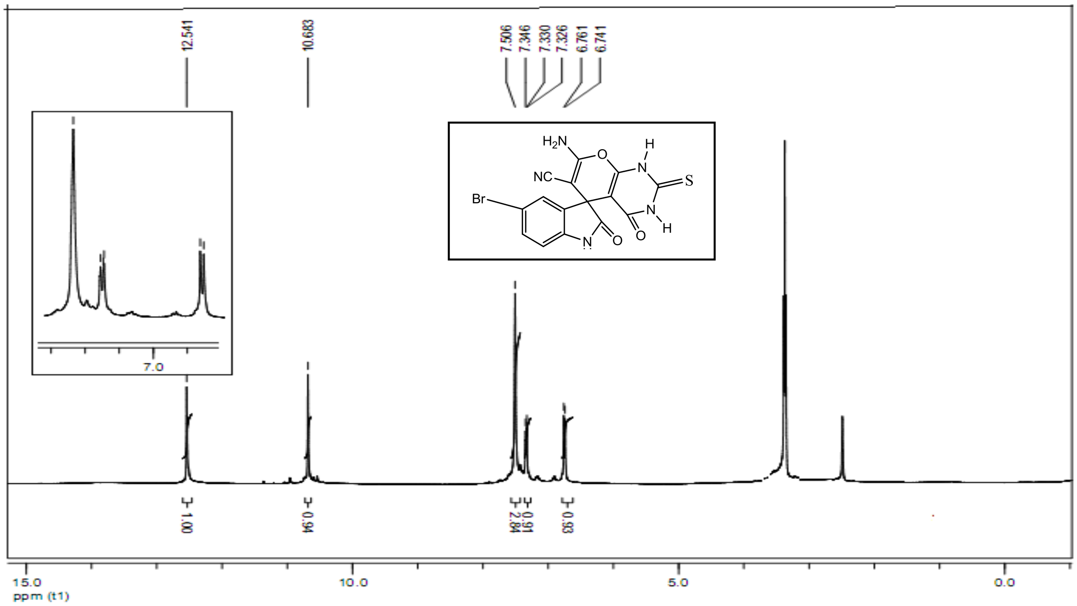

^{1}HNMR of 6d

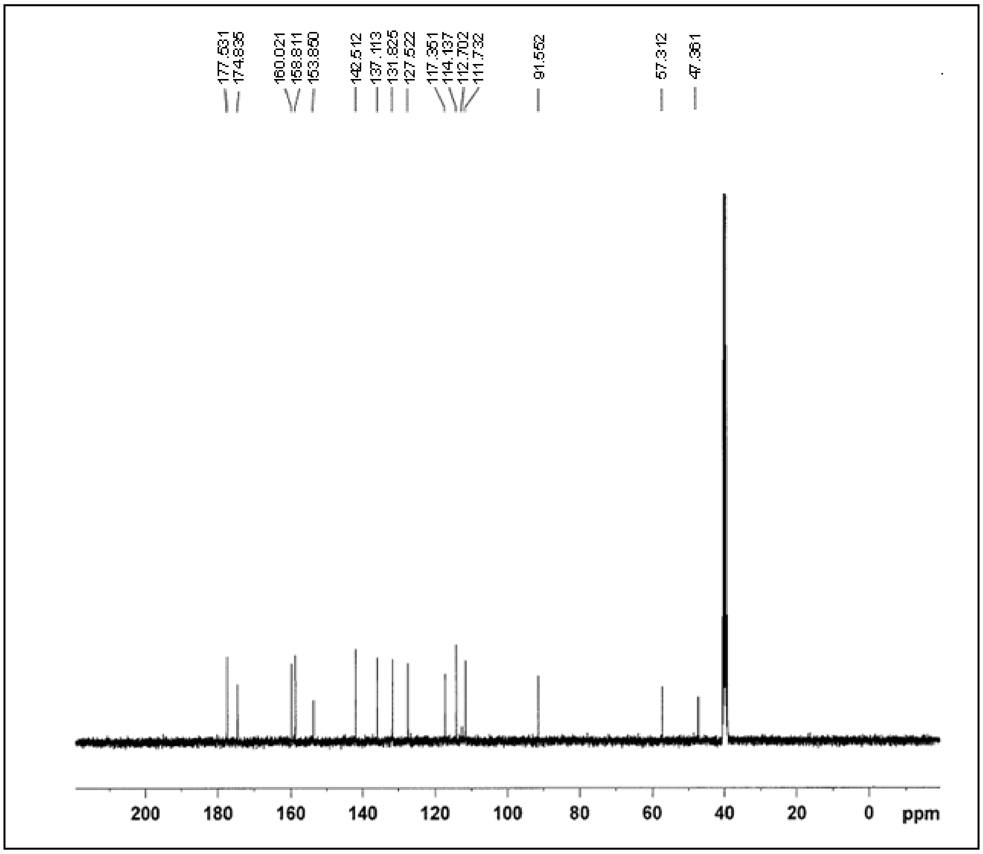

^{13}C NMR of 6d

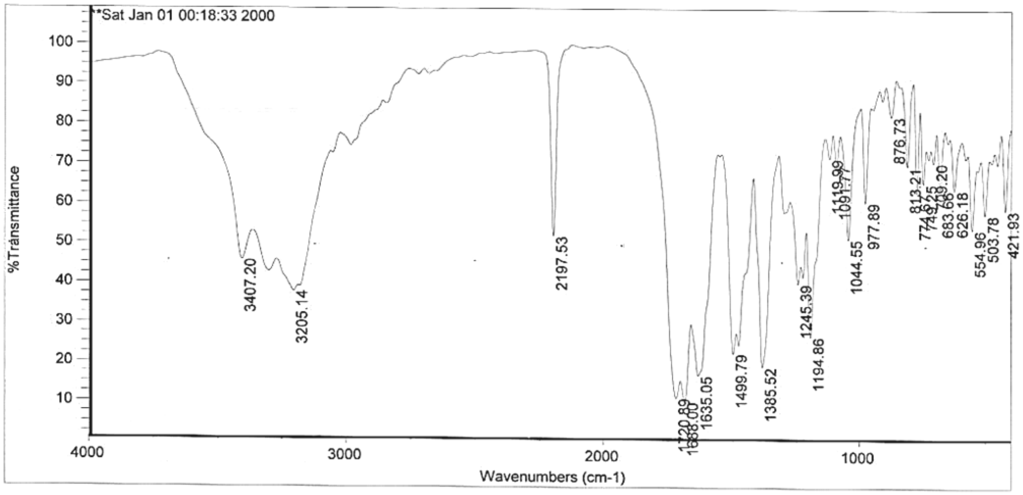

FTIR of 6e

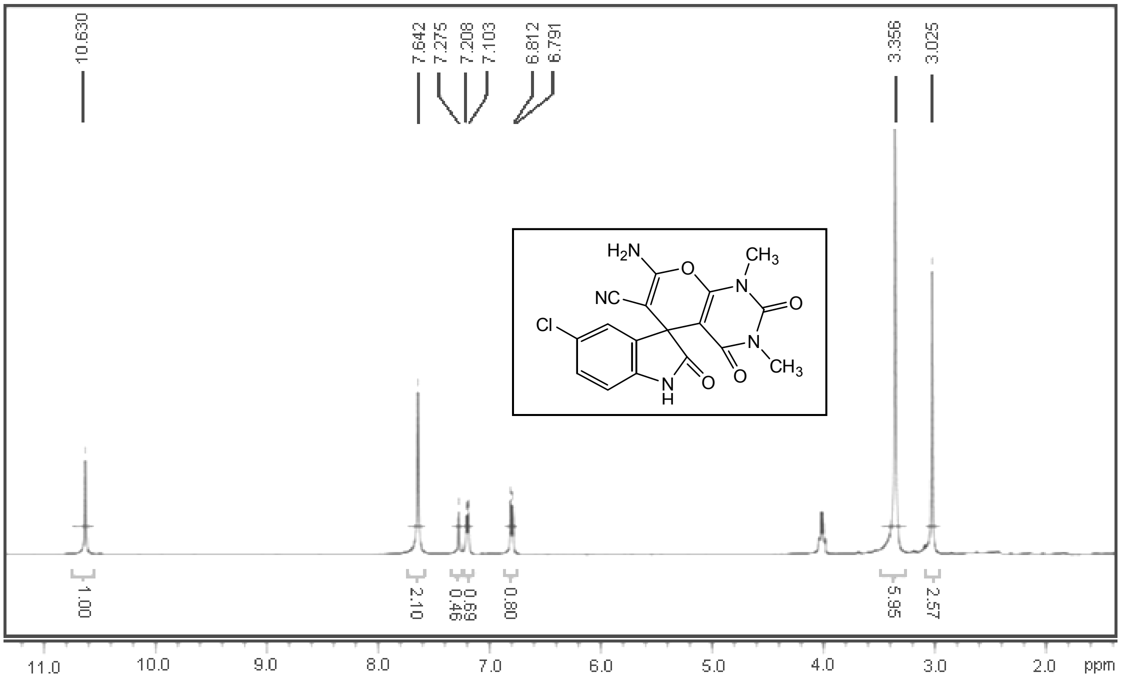

^{1}HNMR of 6e

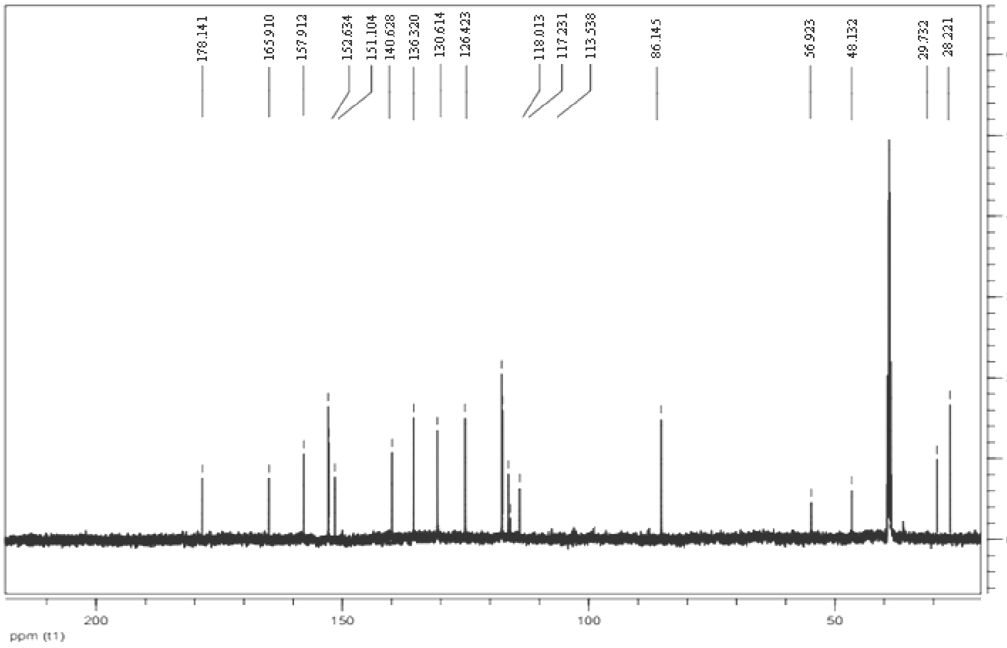

^{13}C NMR of 6e

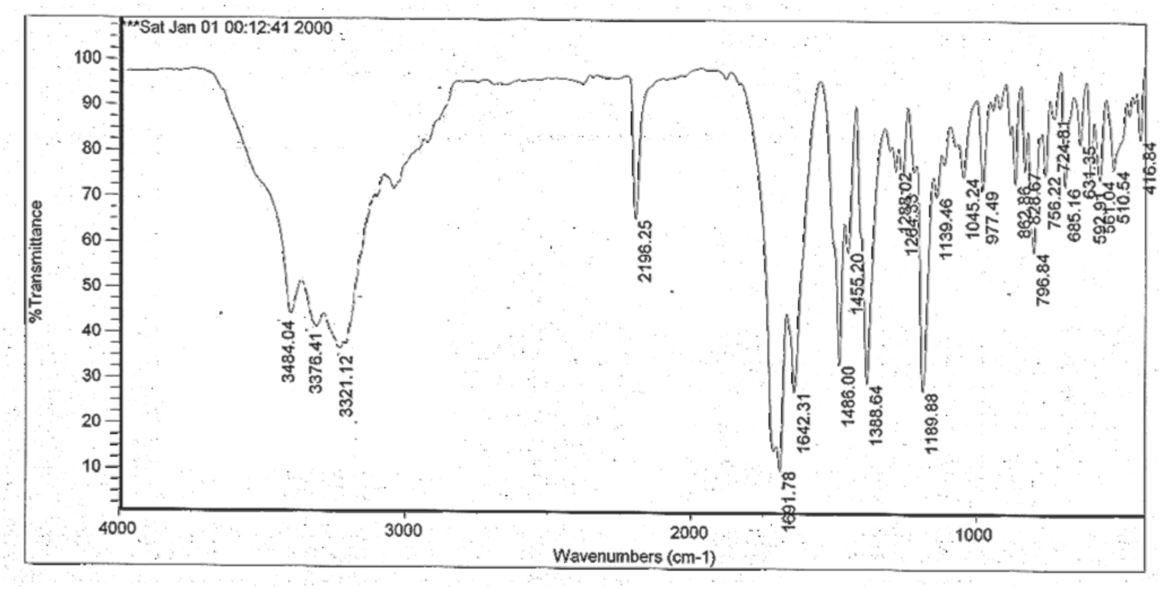

FTIR of 6f

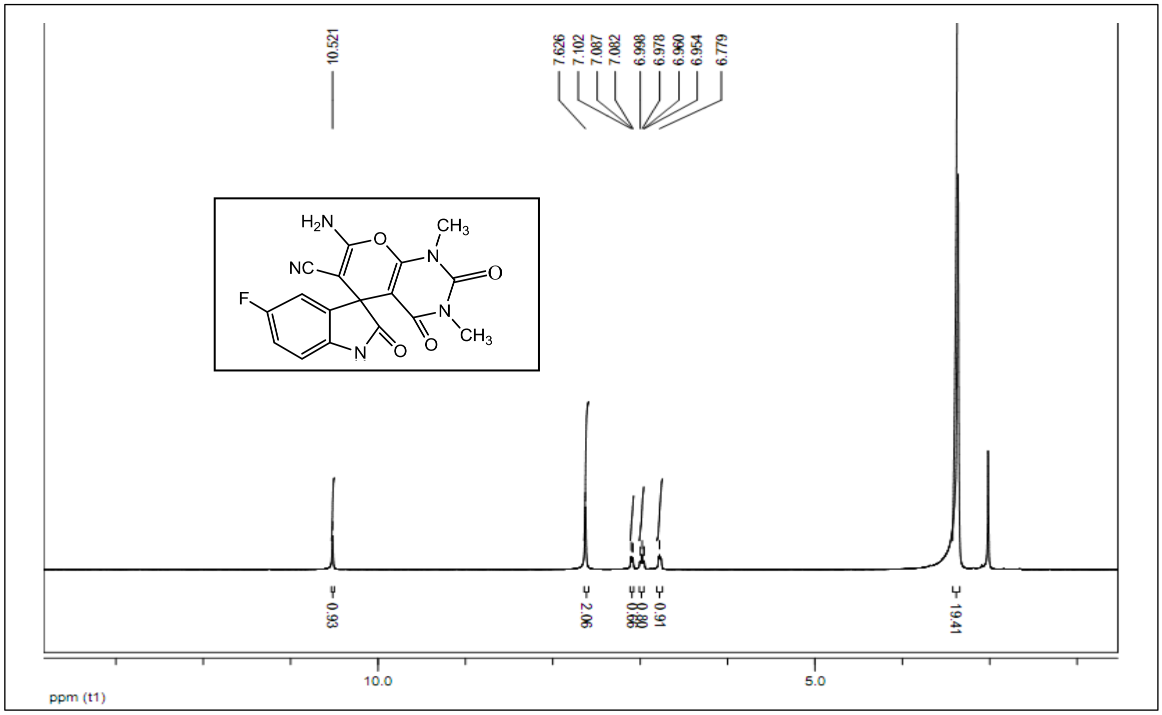

^{1}HNMR of 6f

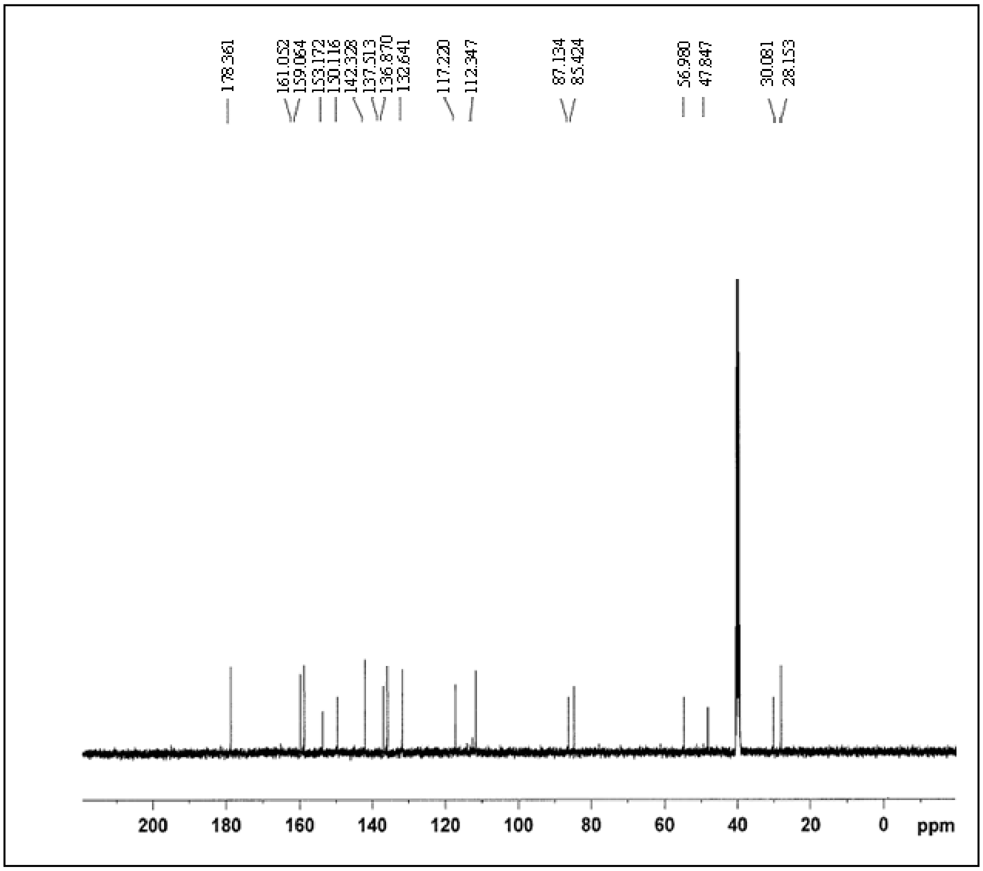

^{13}C NMR of 6f

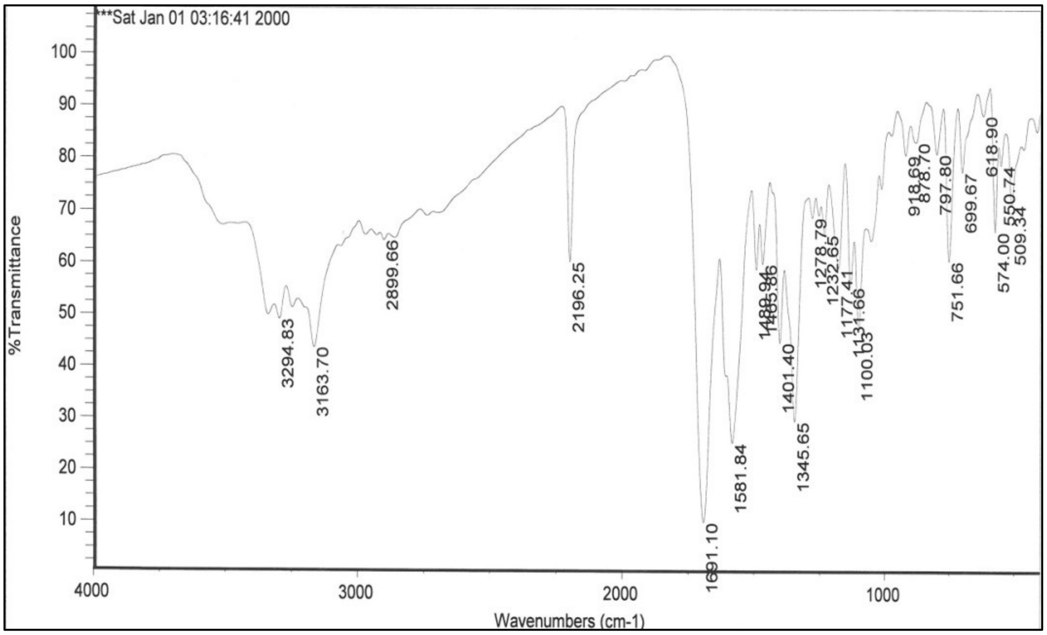

FTIR of 6g

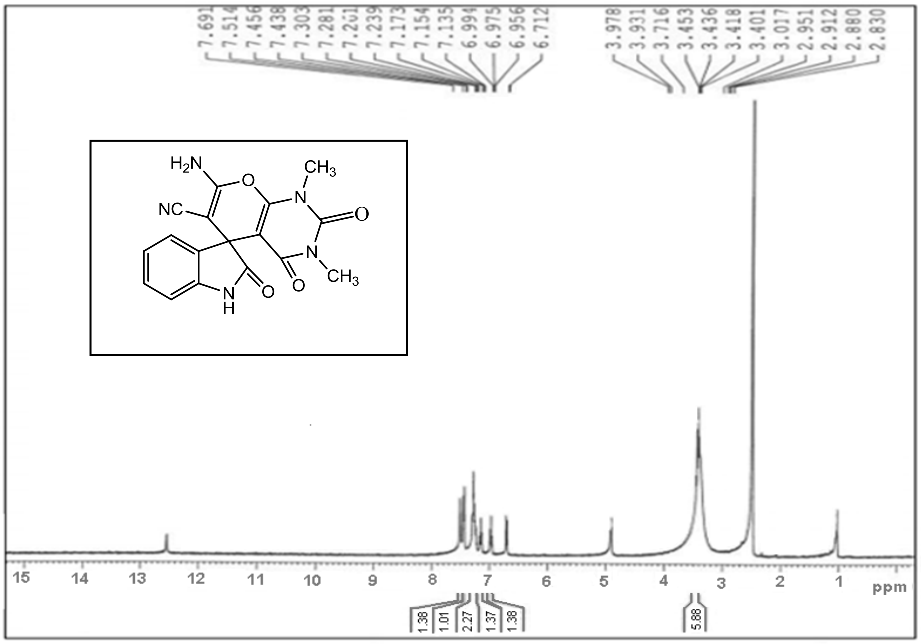

^{1}HNMR of 6g

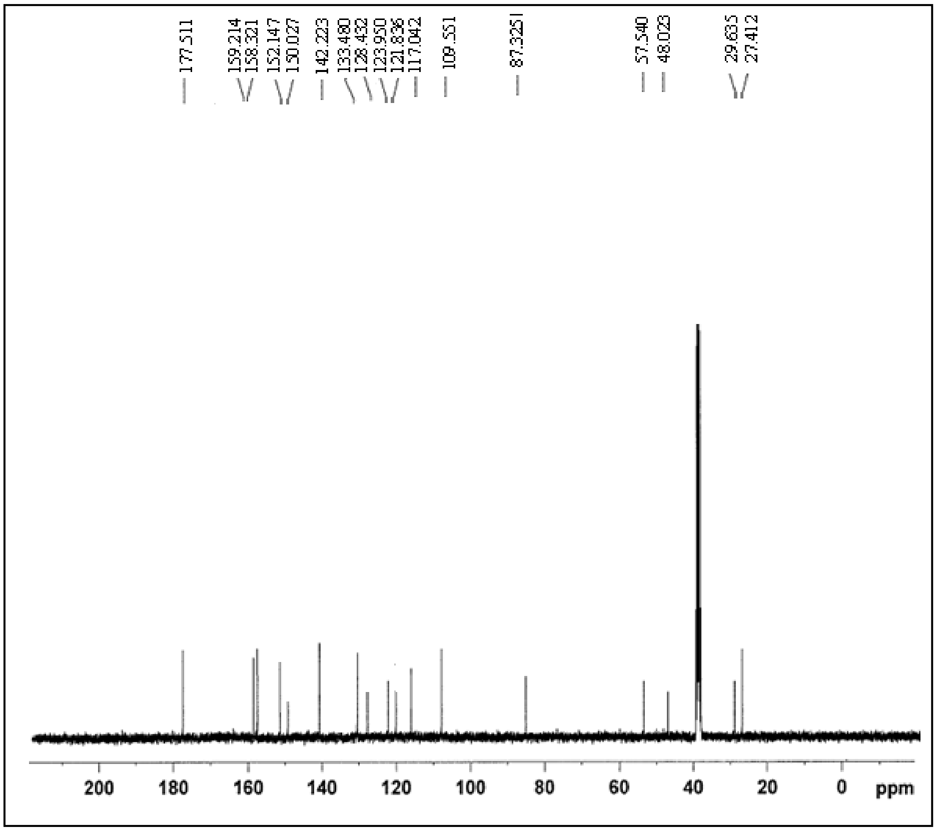

^{13}C NMR of 6g
